# Correlation of homologous recombination deficiency induced mutational signatures with sensitivity to PARP inhibitors and cytotoxic agents

**DOI:** 10.1186/s13059-019-1867-0

**Published:** 2019-11-14

**Authors:** Ádám Póti, Hella Gyergyák, Eszter Németh, Orsolya Rusz, Szilárd Tóth, Csenger Kovácsházi, Dan Chen, Bernadett Szikriszt, Sándor Spisák, Shunichi Takeda, Gergely Szakács, Zoltan Szallasi, Andrea L. Richardson, Dávid Szüts

**Affiliations:** 1 Institute of Enzymology, Research Centre for Natural Sciences, Hungarian Academy of Sciences, Magyar tudosok krt 2, Budapest, H-1117 Hungary; 2 Department of Oncotherapy, University of Szeged, Szeged, Hungary; 3 Department of Medical Oncology, Dana-Farber Cancer Institute, Boston, MA 02215 USA; 4 Center for Functional Cancer Epigenetics, Dana-Farber Cancer Institute, Boston, MA 02215 USA; 5 Department of Radiation Genetics, Kyoto University Medical School, Kyoto, 606-8501 Japan; 6 Institute of Cancer Research, Medical University Vienna, Vienna, Austria; 7 Computational Health Informatics Program (CHIP), Boston Children’s Hospital, Boston, MA USA; 8 Harvard Medical School, Boston, MA USA; 9 Danish Cancer Society Research Center, Copenhagen, Denmark; 10 SE-NAP, Brain Metastasis Research Group, 2nd Department of Pathology, Semmelweis University, Budapest, Hungary; 11 Johns Hopkins University School of Medicine, Baltimore, MD USA

**Keywords:** Mutation signature, BRCA1, BRCA2, RAD51C, PALB2, RAD52, ATM, CHEK2, Microhomology deletion, PARP inhibitor

## Abstract

**Background:**

Homologous recombination (HR) repair deficiency arising from defects in BRCA1 or BRCA2 is associated with characteristic patterns of somatic mutations. In this genetic study, we ask whether inactivating mutations in further genes of the HR pathway or the DNA damage checkpoint also give rise to somatic mutation patterns that can be used for treatment prediction.

**Results:**

Using whole genome sequencing of an isogenic knockout cell line panel, we find a universal HR deficiency-specific base substitution signature that is similar to COSMIC signature 3. In contrast, we detect different deletion phenotypes corresponding to specific HR mutants. The inactivation of BRCA2 or PALB2 leads to larger deletions, typically with microhomology, when compared to the disruption of BRCA1, RAD51 paralogs, or RAD54. Comparison with the deletion spectrum of Cas9 cut sites suggests that most spontaneously arising genomic deletions are not the consequence of double-strand breaks. Surprisingly, the inactivation of checkpoint kinases ATM and CHK2 has no mutagenic consequences. Analysis of tumor exomes with biallelic inactivating mutations in the investigated genes confirms the validity of the cell line models. We present a comprehensive analysis of sensitivity of the investigated mutants to 13 therapeutic agents for the purpose of correlating genomic mutagenic phenotypes with drug sensitivity.

**Conclusion:**

Our results suggest that no single genomic mutational class shows perfect correlation with sensitivity to common treatments, but the contribution of COSMIC signature 3 to base substitutions, or a combined measure of different features, may be reasonably good at predicting platinum and PARP inhibitor sensitivity.

## Background

Somatic mutations in cancer genomes efficiently characterize the DNA repair status of the cancer cells [1–3]. As a consequence, there is much interest in using genomic mutation patterns for the selection of treatments targeted at cells with specific DNA repair defects.

Germline mutations in the genes encoding the homologous recombination (HR) factors BRCA1 or BRCA2 predispose for breast and ovarian cancer [4, 5] and also play roles in the development of prostate, pancreatic, and stomach cancers [6]. The inactivation of *BRCA1* or *BRCA2* due to somatic mutations or *BRCA1* promoter methylation is also observed in these tumor types [7–9]. The *BRCA1/2* status of tumors is relevant for treatment selection, as a *BRCA1/2* defect predicts sensitivity to platinum drugs and PARP inhibitors [7, 10, 11].

Tumors with biallelic inactivation of *BRCA1* or *BRCA2* possess genome-wide somatic single nucleotide variations (SNVs) with a distinct spectrum termed COSMIC signature 3, together with specific but different patterns of short insertions and deletion (indels), and of structural rearrangements [12, 13]. COSMIC signature 3 is one of a set of somatic base substitution signatures identified in cancer genomes [14, 15]. Experiments with isogenic cell lines provided causative evidence for the role of *BRCA1* or *BRCA2* defects in these mutagenic processes [16]. The existence of cancer cases with similar somatic mutation spectra but no *BRCA1/2* mutations [12] raises the possibility that these carry mutations in genes of similar function and therefore may also benefit from treatments designed for *BRCA* mutant cancer [17]. Indeed, whereas only about 30% of ovarian cancer cases have *BRCA* mutations or promoter methylation [18], there exists a cohort of non-*BRCA* mutant ovarian cancer cases with a similar mutation spectrum and mutation burden to *BRCA* mutants [19], and PARP inhibitor treatment was found to be effective in a patient cohort without germline *BRCA* mutation as well [20].

Candidate genes to cause a *BRCA*-like phenotype are those that code for other non-essential factors of the HR pathway: the RAD51 paralogs RAD51B, RAD51C, RAD51D, XRCC2, and XRCC3, which promote the formation of RAD51 subnuclear foci and also can remodel the RAD51 nucleoprotein filament [21, 22]; the binding partners of BRCA1 and BRCA2 including BARD1 [23], CtIP [24], and PALB2 [25]; and further HR factors including RAD54 [26] and RAD52 [27]. Checkpoint proteins involved in signaling the presence of DNA breaks, including ATM and CHK2, may have HR-related roles [28, 29]. Inherited mutations in several of these genes increase the risk of breast or ovarian cancer, including *RAD51C*, *RAD51D*, *BARD1*, *PALB2*, *ATM*, and *CHK2* [30–32]. Consequently, clinical trials are underway to investigate the efficacy of PARP inhibitors on tumors with various subsets of non-BRCA DNA repair gene mutations (trial identifiers: NCT03344965, NCT02286687, NCT03375307).

As an alternative to the search for deleterious gene mutations, a genomic rearrangement-based phenotypic measure of HR defect has been developed that does not incorporate SNVs or short indels. The HRD score predicts response to platinum-containing neoadjuvant chemotherapy in triple-negative breast cancer [33], and also forms part of clinical diagnostic tests for using PARP inhibitors in ovarian cancer [34]. In addition, it has been an open issue whether *BRCA1/2* deficiency/HRD is predictive only of PARP inhibitor/platinum sensitivity or of anthracyclines as well [35, 36]. The incorporation of genomic mutation signatures into the assessment of HR integrity has been suggested [12], and COSMIC SNV signature 3 was found to be associated with *PALB2* and *RAD51C* mutations in breast cancer [3].

The aim of this study was to experimentally determine which HR or checkpoint gene defects cause somatic mutational processes akin to those seen in BRCA1- or BRCA2-deficient cells, and whether the presence of mutational signatures correlates with treatment sensitivity. Using an isogenic knockout cell line panel, we uncovered and analyzed the spontaneous mutagenic processes in HR-deficient cells and demonstrated that the inactivation of checkpoint kinases has negligible mutagenic consequences. SNV mutagenesis showed good but imperfect correlation with PARP inhibitor sensitivity, whereas the mutagenic processes were poor predictors of sensitivity to a range of common cancer cytotoxics.

## Results

### Increased single nucleotide substitution mutagenesis in a range of HR mutant cell lines

**Fig. 1:**
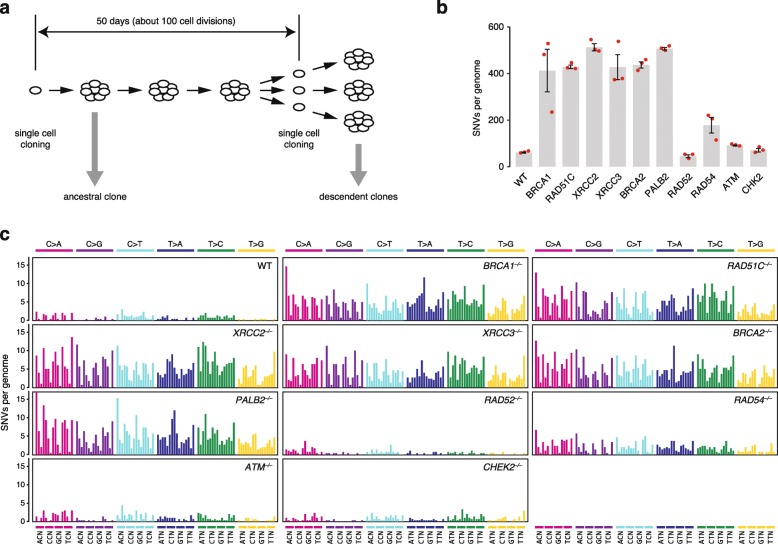
Spontaneous accumulation of SNVs in isogenic cell clones. **a** Experimental scheme. The experiments were started with an ancestral single cell clone. After 50 days of culturing, a further cloning step was performed. Genomic DNA was extracted from each ancestral and descendent clone as soon as a sufficient number of cells were available. **b** The mean number of newly arising SNVs detected per sequenced genome in each indicated cell line. Red symbols show the values for individual samples, error bars indicate standard error of the mean (SEM). **c** Mean spontaneous triplet SNV mutation spectrum in the indicated cell lines. Each mutation class, as indicated at the top of the panel, is separated into 16 categories based on the identity of the preceding and following nucleotide as shown below. The order of the following nucleotides, not shown due to lack of space, is alphabetical

To investigate the genetic dependence of spontaneous mutagenesis, we assembled a collection of isogenic chicken DT40 cell lines harboring homozygous disruptions of each of eight key HR genes plus *ATM* and *CHK2*, which are key components of the DNA damage checkpoint. DT40 lymphoblastoma cells are the only isogenic system currently available to study null mutants in this range of genes, and these cells adequately reproduce human somatic mutagenic processes [16]. The *RAD52*^*−/−*^ cell line was made for this study by deleting a 3323-bp region of the gene including exons 3–7. The derived cell lines were cultured for 50 days between two single cell cloning steps, and DNA was prepared for whole genome sequencing from each ancestral clone as well as three descendent clones per cell line (Fig. 1a, Additional file 1: Table S1). Base substitution mutations and small indels were identified by simultaneously analyzing all sequences together with IsoMut [37]. The mutations detected by IsoMut are unique to one sample, thereby providing an accurate catalog of genetic changes that took place during the experiment (Additional file 1: Tables S2 and S3).

Compared to wild type cells, we observed an approximately seven to eightfold increase in base substitution (SNV) mutagenesis in *BRCA1*^*−/−*^ and *BRCA2*^*−/−*^ cells as shown previously [16]. We found a similarly elevated SNV mutagenesis in cell lines mutant for the *RAD51* paralogs *RAD51C*, *XRCC2*, and *XRCC3*, and in *PALB2*^*−/−*^ mutants (Fig. 1b, Additional file 1: Table S4). The mutation rate was threefold elevated in *RAD54*^*−/−*^ mutants compared to the wild type, and the increase in mutation rate was highly significant in all seven mutants when compared to the wild type (*p* < 0.001, *χ*^2^ test on the summed counts with Bonferroni’s correction for multiple comparison). No increase was seen in *RAD52*^*−/−*^ cells. Surprisingly, we observed only a very moderate elevation of SNV mutation accumulation in the checkpoint-deficient *ATM*^*−/−*^ and *CHK2*^*−/−*^ cells, though the 1.5-fold change in *ATM*^*−/−*^ cells was significant (*p* < 0.001).

### A common base substitution signature describes mutagenesis in all HR-deficient cells

**Fig. 2:**
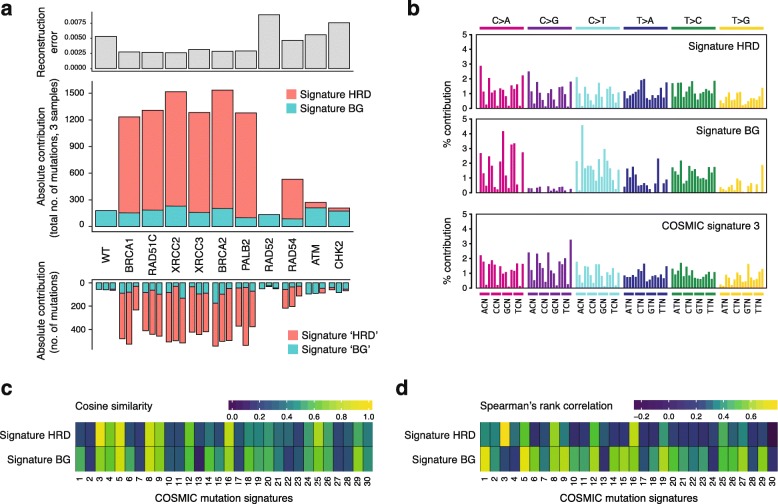
A base substitution signature of HR deficiency. **a** De novo NMF of the detected cell line-specific SNV mutation spectra into two components, termed Signature HRD and Signature BG (middle panel), and the error (root-mean-square deviation) of reconstructing the experimental dataset using these two signatures (top panel). The bottom panel shows the individual SNV counts for each sequenced genome, split using NMF into “HRD” and “BG” signatures that are virtually identical to Signature HRD and BG, respectively. **b** Triplet mutation spectra of Signature HRD and Signature BG shown as the percentage contribution of each triplet mutation type, and COSMIC triplet signature 3 for comparison. **c**, **d** Correlation heat map of the experimentally derived triplet signatures to 30 COSMIC signatures using cosine similarity (**c**) or Spearman’s rank correlation (**d**)

When viewed in the context of the neighboring nucleotides, similar triplet mutation patterns resulted from the disruption of *BRCA1*, *BRCA2*, *PALB2*, the *RAD51* paralogs, and also *RAD54* (Fig. 1c, Additional file 2: Figure S1). We summed the data by cell type and extracted de novo triplet mutation signatures using non-negative matrix factorization (NMF) on the 11 cumulative triplet mutation datasets. A factorization rank of two provided two de novo signatures, which could be used to reconstruct the mutation spectra with small errors (Fig. 2a, Additional file 2: Figure S2, Additional file 1: Table S5). The two signatures resulting from this unsupervised approach appeared to make good biological sense. One signature (termed Signature BG, for background) was similar to the mutation pattern of the wild type, and it was present to a very similar level in all mutants. An additional signature (Signature HRD, for HR deficiency) explained the increased mutagenesis in all cell lines and can therefore be considered a general HR defect-specific triplet mutation signature. The inclusion in the NMF analysis of pre-existing mutations in the ancestral clones provided a very similar HRD-like signature (cosine similarity 0.995) and showed that the experimentally determined ongoing mutation rates do not correlate with past passaging (Additional file 2: Figure S3). Moreover, performing NMF on individual samples also resulted in near-identical HRD-like and BG-like mutation signatures (cosine similarities 0.998 and 0.987, respectively; Fig. 2a, Additional file 1: Table S5). In comparison to COSMIC version 2 signatures, Signature HRD is very similar to signature 3 (cosine similarity 0.940), whereas Signature BG shows more limited cosine similarities, but correlates best with signatures 1 and 5 using Spearman’s rank correlation (Fig. 2b, c, Additional file 1: Table S6). Signature HRD showed best correlation with SBS3 and the new SBS40 in version 3 of the COSMIC signatures (Additional file 2: Figure S4A, B). Interestingly, despite the similarity of signatures 3 and HRD, only 50–60% of mutations in the HR mutant cell lines were explained by signature 3 when attempting a deconstruction with COSMIC version 2 signatures 1, 3 and 5, probably due to the similarity of signatures 3 and 5 (Additional file 2: Figure S4C). Nevertheless, modeling the NMF process on differently sized sample sets drawn from pre-set mixtures of signatures 3 and 5 showed that the number of mutations detected in the HR-deficient cell lines allows the separation of such “featureless” signatures with only 5% mean absolute error (Additional file 2: Figure S5), confirming that SNV mutagenesis in the *BRCA1*, *BRCA2*, *PALB2*, and *RAD51* paralog mutants is indistinguishable both in rate and spectrum.

### Disruption of the HR pathway at different stages gives rise to different short deletion phenotypes

**Fig. 3:**
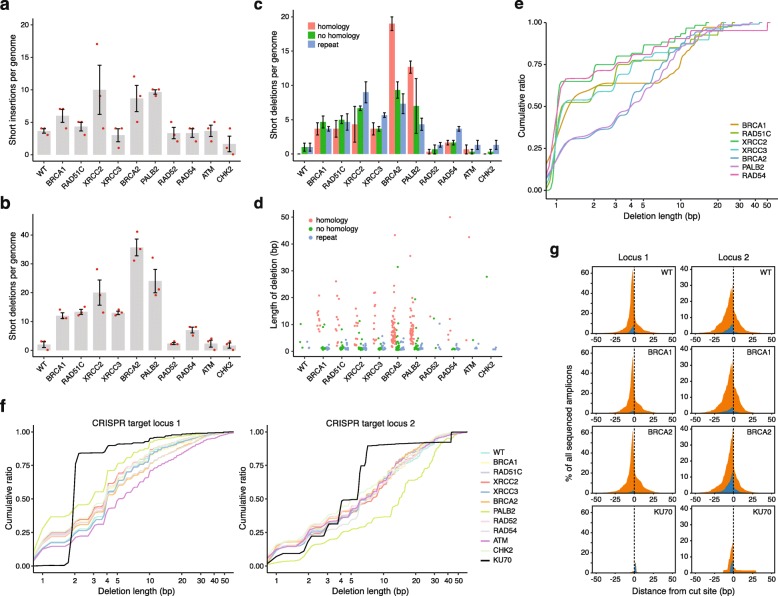
The genetic dependence of the formation of short insertions and deletions. **a**, **b** The mean number of newly arising short insertions (**a**) or short deletions (**b**) generated per sequenced genome in each indicated cell line. Red symbols show the values for individual samples. **c** A classification of detected short deletion events by sequence context. The minimum length of classified microhomologies was 1 bp. Error bars indicate SEM in **a**–**c**. **d** The length of each deletion is shown, color coded according to sequence context. **e** Cumulative size distribution of genomic deletions in those cell lines in which the total number of deletions in the sequenced clones was greater than 10. **f** Cumulative size distribution of deletions at two different CRISPR-targeted genomic loci in each of the indicated cell lines, obtained by amplicon sequencing. **g** Stacked view of all deletions (orange) and insertions (blue) at two CRISPR-targeted loci in four indicated cell lines, shown as the percentage of all sequenced amplicons obtained from genome preparations of cell populations sorted for successful transfection with a plasmid expressing Cas9 and the respective guide RNA

Few short insertions arose in the sequenced genomes, and the difference in the number of short insertions between the wild type and the various mutants was only significant in the case of *XRCC2* (*p* = 0.03, *χ*^2^ test with Bonferroni’s correction) and *PALB2* (*p* = 0.044) (Fig. 3a). In contrast, there was a highly significant (*p* < 0.001) increase of short deletions upon the disruption of *BRCA1*, *BRCA2*, *PALB2*, *RAD51C*, *XRCC2*, or *XRCC3*, and a significant (*p* = 0.039) increase in the *RAD54* mutant. The number of short deletions in *RAD52*^*−/−*^, *ATM*^*−/−*^, and *CHK2*^*−/−*^ cells did not significantly differ from the wild type (Fig. 3b). To better understand how short deletions arise upon defective HR, we classified deletions based on their sequence context (Additional file 1: Table S4). In *BRCA2*^*−/−*^ and *PALB2*^*−/−*^ mutants, whose genomes contained the most deletions, over 50% of deletions showed at least 1 bp of microhomology between the ligated DNA ends (Fig. 3c). In all other HR mutants, deletions with no homology or deletions at repeat sequences were at least as common as the microhomology class. Deletions with microhomology were generally longer than those in the other categories (Fig. 3d) and the cumulative size distribution of all deletions also shows that *BRCA2*^*−/−*^ and *PALB2*^*−/−*^ have a similar phenotype that is distinct from those of other HR mutants (Fig. 3e). This phenotype of more, larger deletions with more common microhomologies is presumably connected to a joint function of the BRCA2/PALB2 complex. The phenotypes of the RAD51 paralogs were similar to each other; *RAD54*^*−/−*^ had a weaker deletion phenotype, whereas *BRCA1*^*−/−*^ displayed a surprising lack of short deletions in the 2–7-bp size range. In agreement with this analysis, a classification of indels into COSMIC version 3 short indel signatures [38] found the microhomology deletion dominated signature ID6 mainly in the *BRCA2*^*−/−*^ and *PALB2*^*−/−*^ datasets (Additional file 2: Figure S6). Thus, unlike in the case of SNVs, the disruption of the HR pathway at the level of different participating proteins results in distinct patterns of short genomic deletions.

### Spontaneously arising very short deletions are not the consequence of double-strand break repair

To understand whether the different deletion phenotypes are caused by the influence of a partially disrupted HR pathway on DNA double-strand break (DSB) repair, we investigated the spectrum of mutation events at CRISPR/Cas9-induced blunt ended DSBs at two different genomic loci using amplicon sequencing. The majority of mutation events were deletions, alongside some insertions and some mixed events (Additional file 2: Figure S7). Unlike in the case of spontaneous deletions where deletion sizes varied, the size distribution of Cas9 DSB-induced deletions was very similar across all investigated HR mutants and the wild type cell line and resembled the spontaneous genomic deletion phenotype of *BRCA2* and *PALB2*, with a median deletion size of approximately 4 bp (Fig. 3f). In contrast, we observed a reduction in the number and size of deletions in a *KU70*^*−/−*^ mutant cell line (Fig. 3f, g, black line), demonstrating the role of non-homologous end joining in shaping the outcome of DSB repair. The majority of spontaneous deletions observed in HR mutants are thus shorter than those observed at nuclease-induced DSBs. With the caveat that the Cas9 cut does not reproduce all types of potential spontaneous DSBs, these results suggests that the very short deletions are not the consequence of DSB repair but of a different process that is upregulated in the absence of HR proteins.

### Patterns of large-scale rearrangements differentiate HR mutants

**Fig. 4:**
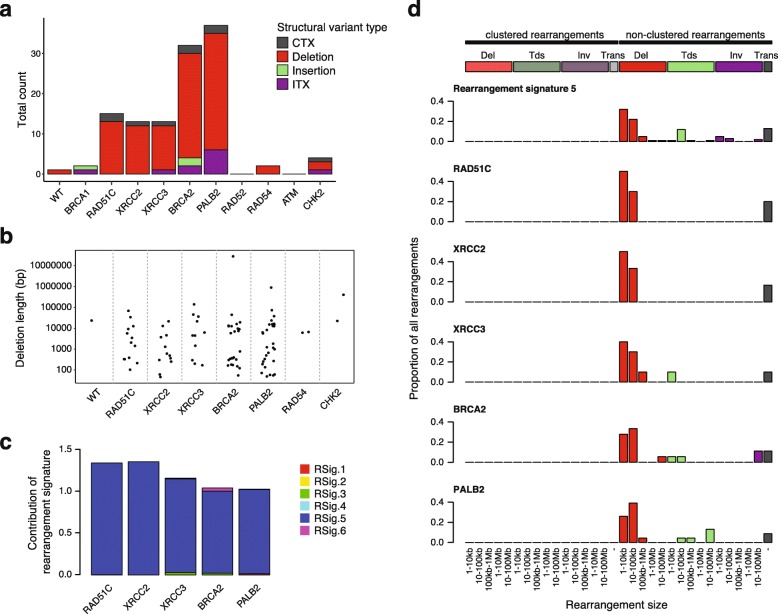
Classification of large-scale rearrangements. **a** Total count of large-scale rearrangements in three sequenced genomes per cell line, classified by CREST as interchromosomal translocation (CTX), intrachromosomal translocation (ITX), deletion, or insertion. **b** The size distribution of large deletions. **c** Reconstitution of the rearrangement profile of cell lines with > 10 events using 6 rearrangement signatures (Rsig). **d** Classification of rearrangements in the same cell lines according to categories used for the definition of rearrangement signatures [12]. Rearrangement signature 5 is shown for comparison

When searching for structural variations, we found a predominance of large deletions of a wide size range in *BRCA2*^*−/−*^ and *PALB2*^*−/−*^ cells, as well as in the three tested *RAD51* paralog mutants (Fig. 4a, b, Additional file 1: Tables S4 and S7). The rearrangement phenotypes in these five cell lines could be best reconstructed with rearrangement signature 5 defined from the analysis of breast cancer genomes (Fig. 4c) [12]. Indeed, the size distribution of deletions and the presence of a few other types of non-clustered rearrangements in *RAD51C*^*−/−*^, *XRCC2*^*−/−*^, *XRCC3*^*−/−*^, *BRCA2*^*−/−*^, or *PALB2*^*−/−*^ mutant cells closely resembled the predominant rearrangement signature of *BRCA2*-deficient breast tumors (Fig. 4d).

### HR-deficient cells show differential sensitivities to cancer cytotoxics

**Fig. 5:**
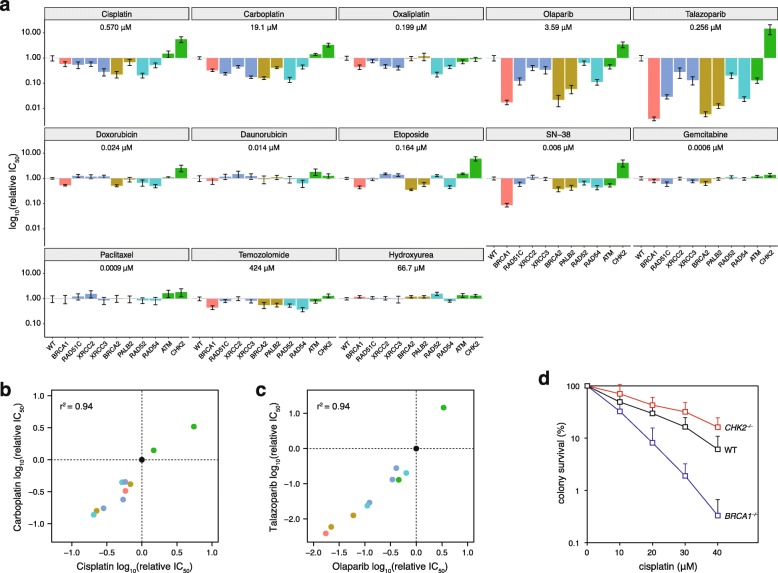
Sensitivity of HR-deficient cell lines to cytotoxic agents. **a** Results of cytotoxicity measurements shown as the fold difference in sensitivity to the indicated drugs of each mutant cell line as compared to the wild type. The mean relative IC_50_ values and SEM of three independent measurements are shown. The mean absolute IC_50_ value of the wild type line is shown for each drug. **b**, **c** Correlation of the relative IC_50_ values of cisplatin vs. carboplatin (**b**) and olaparib vs. talazoparib (**c**). Each marker represents a cell line, color coded as in **a**; the wild type is shown in black. The *r*^2^ value for simple linear regression is shown. **d** Colony survival assay following the treatment of the indicated cell lines with cisplatin for 1 h. The mean survival percentages and SEM of three independent measurements are shown

The main incentive to investigate the mutagenic processes arising from the defect of a range of HR genes is to determine whether the observed mutation patterns may be predictive of therapeutic effect. To link mutational signatures to therapy, we measured the sensitivity of each experimental cell line to a wide range of commonly used cancer therapeutics (Fig. 5a, Additional file 1: Tables S8 and S9). The greatest hypersensitivity was seen in the case of the PARP inhibitors olaparib and talazoparib. Most HR mutant lines were also hypersensitive to platinum agents. A few cell lines, primarily *BRCA1*^*−/−*^, *BRCA2*^*−/−*^, and *PALB2*^*−/−*^, were also slightly sensitive to the anthracyclin doxorubicin, the topoisomerase II inhibitor etoposide, the topoisomerase I inhibitor SN-38 (the active form of irinotecan), and the alkylating agent temozolomide. *RAD51* paralog mutants were also sensitive to PARP inhibitors, but to a lesser extent than the *BRCA1/2* mutants, and showed no sensitivity to topoisomerase inhibitors. Interestingly, *RAD52*^*−/−*^ mutants were most sensitive to platinum agents, whereas *RAD54*^*−/−*^ mutants showed specific sensitivity to PARP inhibitors. The disruption of the DNA damage checkpoint in *ATM*^*−/−*^ mutants did not sensitize cells to most tested agents. Even more unexpectedly, *CHK2*^*−/−*^ mutant cells were less sensitive to platinum agents, PARP inhibitors, and topoisomerase inhibitors than the wild type line. We confirmed the reduced sensitivity of *CHK2*^*−/−*^ cells to cisplatin, olaparib, and etoposide using a colony survival assay with short treatment duration to exclude the possibility that this result was influenced by potentially slower growth under the conditions of the cytotoxicity assay (Fig. 5d, Additional file 2: Figure S8).

The reliability of the cytotoxicity assay is highlighted by the excellent concordance of sensitivities to drugs with common mechanisms (Fig. 5b, c) and by the lack of sensitivity to drugs that do not cause DNA damage or target DNA repair, such as paclitaxel and hydroxyurea. The different pattern of sensitivities to oxaliplatin as compared to cisplatin and carboplatin supports findings that oxaliplatin kills cells through additional mechanisms independent of the DNA damage response [39]. Minimal sensitivities were seen to anthracyclins, restricted to a slight doxorubicin sensitivity of *BRCA1*, *BRCA2*, and *RAD52* mutants.

### Mutation patterns in cancer genomes with HR gene defects

**Fig. 6:**
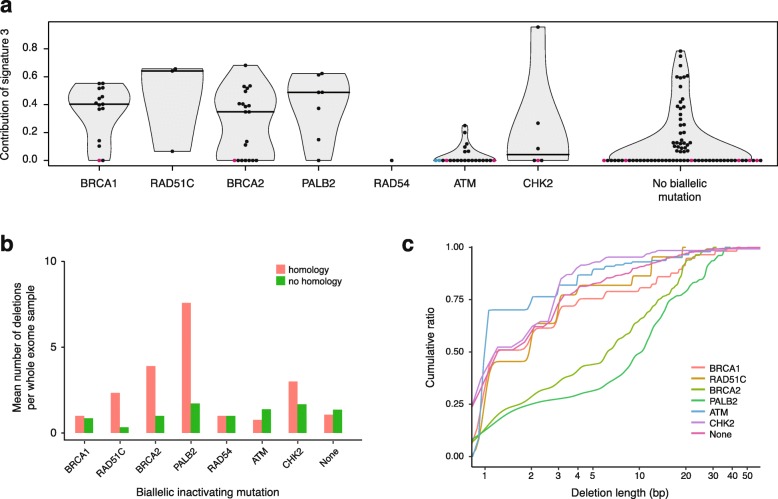
Mutational features of HR-deficient tumor genomes. **a** The contribution of COSMIC signature 3 to the SNV spectrum derived from whole exome sequence data of tumors from various tissues bearing biallelic inactivating mutations in the indicated genes. As a control, an equal randomly selected set of whole exome datasets was used with the same tissue type distribution and no biallelic mutation in the analyzed genes. A horizontal line indicates the median. Samples with over 40% of SNVs belonging to COSMIC spectra 6, 15, 20, or 26 suggesting mismatch repair deficiency are shown in magenta; samples with over 40% of SNVs belonging to COSMIC spectrum 11 suggesting *POLE* defect are shown in cyan. **b** Classification of deletions in the same samples by sequence context as on Fig. 3c; apparent deletions at repeats are not shown as these appear to include many false positive mutation calls. **c** Cumulative size distribution of deletions in those mutants in which the total number of deletions in all samples was greater than 10. Samples with suspected mismatch repair or *POLE* defect based on the SNV spectrum deconstruction were omitted from **b**, **c**

We were able to detect specific mutational processes in genomes of isogenic cell line models with disrupted HR genes, but not in *ATM*^*−/−*^ or *CHK2*^*−/−*^ mutant cells. To test the utility of the mutation patterns in identifying cancers with defects in the same HR genes, we re-investigated a panel of tumor whole exome sequences in which cases of biallelic inactivation of HR-related genes were identified (Additional file 1: Table S10) [40]. In addition to *BRCA1* and *BRCA2*, most tumors with biallelic inactivation of *RAD51C* or *PALB2* showed a high contribution of COSMIC signature 3 to the total exomic somatic SNV load (Fig. 6a). In further agreement with the cell line-based data, the majority of samples with *ATM* or *CHK2* biallelic inactivation showed zero or very low contribution of signature 3. The median contribution of signature 3 to a matched random TCGA sample set with no inactivating mutation in the genes investigated in this study was zero, but there was a considerable number of mostly ovarian cancer samples which nevertheless contained mutations assigned to signature 3 (Fig. 6a, Additional file 2: Figure S9). Using the entire signature set from COSMIC version 3 resulted in lower sensitivity but higher specificity of detecting signature SBS3 in HR mutant samples (Additional file 2: Figure S10). The number of short (< 50 bp) deletions showed no obvious differences between the various genotypes. These were dominated by 1 bp deletions at repeat sequences, some of which we suspect to be false mutation calls (Additional file 1: Table S10). However, there was a clear and specific increase of deletions with microhomology in *BRCA2* and *PALB2* mutant samples (Fig. 6b), and the cumulative size distribution of deletions also separates the *BRCA2* and *PALB2* phenotypes from the others (Fig. 6c), in remarkable agreement with the cell line-based data. These results confirm the cell line-based findings, suggesting that the contribution of signature 3 to the SNV load can be a useful biomarker for identifying tumors with *RAD51* paralog or *PALB2* loss-of-function alongside those with *BRCA1* or *BRCA2* defects, whereas deletions with microhomology identify *BRCA2* and *PALB2* defects.

## Discussion

In this study, we used isogenic cell lines to detect similarities and differences in the patterns of spontaneous genomic mutagenesis resulting from the disruption of a range of HR genes, and also demonstrated that the inactivation of the DNA damage checkpoint does not give rise to a phenotype indicative of HR deficiency. A dataset derived from cytotoxicity measurements of common cancer therapeutics promotes further investigation for the evaluation of the predictive value of different genomic mutational signatures.

Three main types of mutational features are available for correlation with treatment sensitivity: base substitutions, short indels, and large rearrangements. Six of the tested mutants showed high rates of SNV mutagenesis characterized by signature 3, and *RAD54*^*−/−*^ cells had a similar, more moderate phenotype. According to the cytotoxicity results, the contribution of signature 3 is therefore a reasonable overall predictor of PARP inhibitor and platinum sensitivity. However, the lower sensitivity of *RAD51* paralog mutants compared to *BRCA1*^*−/−*^, *BRCA2*^*−/−*^, and *PALB2*^*−/−*^ suggests a lower predictive value of SNV signatures for PARP inhibitor treatment. Deletion patterns can most accurately identify defects of *BRCA2* and *PALB2*, also confirmed in tumor genomes; therefore, a high proportion of deletions with microhomologies may be a good predictor of treatment with PARP inhibitors, cisplatin or carboplatin. Structural variations with rearrangement signature 5 specifically identify cells with disfunctional *BRCA2*, *PALB2*, or *RAD51* paralogs; therefore, their presence also correlates with sensitivity to PARP inhibitors and platinum.

There are two caveats with using the investigated genomic features for prediction of drug sensitivity. False positives may arise due to different sensitivities of cell lines with the same genomic features, e.g., in the case of *RAD51C* versus *BRCA2* mutants. False negatives arise due to the lack of certain genomic features in sensitive cell lines, as in the case of the lack of signature 5 rearrangements in *BRCA1* mutant cells, which are nevertheless very sensitive to PARP inhibitors. The use of a linear combination of the strength of mutational processes, and the incorporation of a rearrangement signature characteristic of *BRCA1* mutant cells led to an improved classification of *BRCA1/2* deficiency, named HRDetect [41]. The setup of our cell line-based experiments did not produce a sufficient number of large rearrangement events to meaningfully calculate HRDetect scores, but the results suggest that HRDetect will successfully identify cells and tumors with defects in *PALB2*, *RAD51* paralogs, or *RAD54*. Whereas this approach can lead to the reduction of false negatives, the problem of false positives may be inherent to the different mechanistic roles of the investigated proteins in the HR pathways.

Genomic deletion data may help understanding the distinct functions of HR proteins. The majority of deletions in wild type cells are very short, with a median length of 1 bp. The same is true in *BRCA1*, *RAD54*, and *RAD51* paralog mutants, though an appearance of longer deletions with microhomology is also seen. In contrast, longer deletions with microhomology dominate in *BRCA2* and *PALB2* mutants, and these closely resemble the spectrum of deletions at Cas9-induced DSBs in wild type and HR mutant cells. It therefore seems that HR proteins function in preventing two distinct mutagenic processes that give rise to genomic deletions. A replicative process may give rise to the very short (1–2 bp) deletions as well as SNVs with an HRD spectrum, and all investigated HR proteins antagonize this apart from RAD52. The more moderate SNV and deletion phenotype of *RAD54* may simply be due to redundancy with *RAD54B* [42]. Conversely, DSB repair by non-homologous end joining is likely to produce the observed longer (3–20 bp) deletions with microhomology. The results therefore suggest that BRCA2 and PALB2 have a distinguished role in preventing the formation of spontaneous DSBs, possibly through protecting stalled and reversed replication forks from nucleolytic cleavage [43, 44].

The near-complete lack of mutagenic consequences of *ATM* or *CHK2* disruption is surprising and suggests that the main function of this DNA damage checkpoint mechanism is not the facilitation of correct repair, at least in cells with a moderate amount of endogenous DNA damage. This is in agreement with reports showing no impairment of HR repair upon CHK2 inactivation [45, 46]. Indeed, our results and further recent data did not show an increased contribution of signature 3 mutations to somatic SNVs in ATM-deficient tumors [47, 48]. Instead, the main relevance of the pathway to tumor cells may be cell cycle arrest and p53-mediated apoptosis, explaining the mutual exclusivity of *ATM* and *TP53* inactivation in breast cancer [47]. The *ATM* or *CHK2* mutant cell lines typically also did not show sensitivity to DNA damaging agents and PARP inhibitors, and despite promising earlier results, olaparib did not lead to improved overall survival for patients with ATM-negative gastric tumors in a phase 3 trial [49, 50]. Taken together, the mutagenicity and drug sensitivity results argue against making a connection between HR-deficient and ATM/CHK2-deficient cancers.

## Conclusion

In conclusion, our results suggest that no single genomic mutational class shows perfect correlation with sensitivity to common treatments, but the contribution of COSMIC signature 3 to SNVs, or a combined measure of different features, may be reasonably good at predicting platinum and PARP inhibitor sensitivity. The mutagenic processes result from loss of integral HR pathway components; therefore, cancers with non-*BRCA* HR defects, such as germline or somatic mutations in *PALB2*, *RAD51B*, *RAD51C*, or *RAD51D*, or their inactivation through promoter methylation [3], would also receive a positive treatment prediction. However, if the tested cell lines accurately model the relevant tumor tissues, the lower sensitivity of *RAD51* paralog mutants to PARP inhibitors may predict a reduced clinical response in tumors with the corresponding gene defects. The employed p53-negative lymphoid cell line model clearly has limitations [51, 52], but the multiple levels of agreement between cell line and tumor-derived mutation patterns encourage the further use of isogenic cell line models to decipher the complex mutational processes in human cancer.

## Methods

### Cell culture

The following DT40 cell lines were used: wild type, *BRCA1*^*−/−*^, and *BRCA2*^*−/−*^ as used in [16]; *PALB2*^*−/−*^ [53]; *RAD51C*^*−/−*^, *XRCC2*^*−/−*^, and *XRCC3*^*−/−*^ [22]; *RAD54*^*−/−*^ (gene name *RAD54L*) [54]; *ATM*^*−/−*^ [55]; and *CHK2*^*−/−*^ (gene name *CHEK2*) [56]. *RAD52*^*−/−*^ mutant cells were generated by homologous gene targeting, replacing the genomic region from the NdeI restriction site upstream of exon 3 until the BamHI restriction site downstream of exon 7 on each allele with blasticidin and hygromycin selection cassettes, respectively. All cell lines and all gene mutations were verified using the whole genome sequence data. Cells were grown at 37 °C under 5% CO_2_ in RPMI-1640 medium supplemented with 7% fetal bovine serum, 3% chicken serum, and 50 μM 2-mercaptoethanol. PX458 (Addgene plasmid #48138) [57] transiently expressing Cas9, GFP, and a guide RNA was transfected into 600,000 cells using a Nucleofector 4D instrument (Lonza) with program CN-150. GFP+ cells were sorted 24 h later, and after a further 24 h, by which time the arising mutation spectrum is expected to be stable [58], the regions of the targeted loci were amplified from genomic DNA preparations using indexed PCR primers.

### DNA sequencing and mutation calling

Whole genome sequencing on Illumina HiSeq X Ten instruments (2 × 150 bp paired end) to 30 GB coverage per cell clone was done at Novogene, Beijing, China. A mean coverage of 27× was achieved in the near-diploid DT40 genome [52]. The alignment of reads was done as described, using the Galgal4.73 reference genome (16). Independently arising SNVs and short indels were identified using IsoMut, run on all samples together with default settings [37]. The output was post-filtered such that no more than five SNVs and one indel were detected in the ancestral clones, as mutations detected as unique in these samples provide an internal control for false positives. To identify mutations in each ancestral clone that formed since the last common ancestor with the other cell lines, IsoMut was re-run with the omission of the descendent clones of the respective genotype. Mutations in protein coding sequences of ancestral clones are presented in Additional file 1: Table S11. Short deletions were classified as repeat if the deleted sequence was present in at least two tandem copies and as microhomology if the sequence at the two breakpoints contained at least 1 bp of homology. Structural variations were detected using CREST [59] with post-filtering steps. The post-filtering of the CREST output utilized three filters to deem a structural event valid: both breakpoints had to be unique among the samples, both breakpoints had to be covered by reads in all of the samples, and the structural variant had to be supported by at least five soft-clipped reads. CRISPR-targeted amplicons were sequenced on Illumina HiSeq 2500.

### Signature analysis

Individual SNV spectra were averaged for each genotype. De novo NMF decomposition and fitting of triplet signatures was performed using the R package *MutationalPatterns* [60]. During de novo NMF of experimental data, an optimal component number of two was chosen based on the cophenetic correlation coefficient and the residual sum of squares values (Additional file 2: Figure S2) and the same settings were used on simulated data (Additional file 2: Figure S5). Signature deconstruction was performed using the *deconstructSigs* R package [61] using version 2 of the COSMIC signatures [15] unless otherwise stated. For comparisons to COSMIC triplet signatures, DT40 triplet signatures were adjusted by multiplying with the ratio of triplet occurrences in the human and chicken genomes [62]. COSMIC version 3 short indel classification (Additional file 1: Table S12) was performed using a custom script that used the IsoMut indel output list (Additional file 1: Table S3) as its input. For the structural variation signature analysis, we used the rearrangement signature set defined by [12]. Potential clustered events were detected using the R package *copynumber*, using a piecewise constant fitting method with parameters *k*_min_ = 10 and *γ* = 25. After classifying the indels and the structural variants, the signature contributions were calculated by non-negative least squares regression using the R package *pracma*.

### CRISPR-based DSB repair analysis

Two loci were targeted: exon 6 of the *HMBS* gene (gRNA directed against chr24:40917-40636) and exon 1 of the *XPC* gene (gRNA directed against chr12:10787326-10787346). The gRNA sequences targeting locus 1 and locus 2 were GCACCAATGGTAAAGCCAGG and GATCTGCTCGCCGCTATGGCG, respectively. To amplify the DNA in the region surrounding the targeted sites, we used primers gacNNNNNNCCACACTGCAAAACATTAAGTCC and gacNNNNNNCTGTTCAGTGTTGTGACTGC for locus 1 and gacNNNNNNGTCCGCCATCTTTCAAACC and gacNNNNNNCCGGGCCGCCTTTTGC for locus 2, where underlined letters denote flanking sequences and N’s denote sample-specific barcodes. The amplicon sequencing reads were first preprocessed with Trimmomatic [63] to remove Illumina sequencing adapters and sequences of low base quality. The read pairs were merged using FLASH2 [64] with maximal overlap length set to 150 and maximal mismatch density set to 0.05. The merged amplicons were aligned against the theoretical amplicon sequence using the global pairwise aligner Needle from the EMBOSS toolset [65] and the sequence alterations found in each amplicon were summarized and visualized with a custom Python script.

### Sensitivity measurements

For cytotoxicity assays, 1000 cells per well in 384-well plates were incubated with cytotoxic drugs (Additional file 1: Table S9) at a range of concentrations. Cell viability was measured after 72 h using PrestoBlue (Thermo Fisher) and an EnSpire plate reader (Perkin-Elmer). Three technical replicates were averaged per experiment. Data were normalized to untreated cells; curves were fitted with the GraphPad Prism software using the sigmoidal dose–response model. Curve fit statistics were used to determine IC_50_ values. Colony survival assays were performed by plating cell dilutions in medium containing 1% methylcellulose following 1-h cisplatin treatments or 24-h treatments with olaparib or etoposide, and counting the emerging cell colonies.

### Analysis of TCGA samples

Whole exome sequencing data of samples from a wide variety of tissues were obtained based on the pipeline described by Riaz and colleagues [40] from the Broad GDAC Firehose portal. Samples with fewer than 100 somatic SNVs were excluded from analysis. Mutational annotation format files were processed with the R software environment. For each investigated HR-related gene, only samples with germline or somatic biallelic pathogenic mutations, either with loss of heterozygosity or with compound heterozygosity, were considered as confirmed pathogenic variants. Samples with only monoallelic pathogenic mutations or with variants of uncertain significance but no confirmed pathogenic mutations were excluded. For negative control, samples with neither pathogenic mutations nor variants of uncertain significance in any of the investigated genes were selected. Deconstruction of mutational signatures to the 30 COSMIC signatures was performed with the *deconstructSigs* R package [61].

## Supplementary information

**Additional file 1: Table S1.** Provides general sequencing statistics. **Tables S2** and **S3.** Contain a catalogue of all detected SNVs and indels, respectively. **Table S4.** Contains a summary of all detected genetic alterations. **Table S5.** Provides the numerical values of de novo SNV signatures. **Table S6.** Contains numerical data for cosine similarity and Pearson correlation values between SNV signatures. **Table S7.** Lists all detected and post-filtered SVs. **Table S8.** Provides all measured IC50 doses on all cell lines. **Table S9.** Lists the sources of all chemotherapeutic drugs used in this study. **Table S10.** Provides the identifier, genotype, mutation data and SNV signature contributions of analysed TCGA samples. **Table S11.** Contains a catalogue of all detected coding SNVs in the ancestral clones. **Table S12.** Contains aggregated indel profiles according to PCAWG nomenclature.

**Additional file 2: Figure S1.** Shows SNV spectra normalised to the frequency of each triplet occurrence. **Figure S2.** Shows the NMF output used for defining signatures HRD and BG. **Figure S3.** Shows the derivation of SNV signatures on a dataset that includes pre-existing mutations in the ancestral clones. **Figure S4.** Correlates detected SNV spectra and signatures with COSMIC SNV signatures. **Figure S5.** Shows a simulation of separating ‘featureless’ SNV signatures. **Figure S6.** Shows a deconstruction of detected indel datasets into indel signatures. **Figure S7.** Shows the results of amplicon sequencing across Cas9 cut sites. **Figure S8.** Shows drug sensitivity measurements on ancestral and descendent clones. **Figures S9** and **S10.** show the contribution to the SNV load of TCGA samples of COSMIC v2 and v3 SNV signatures, respectively.

**Additional file 3.** Review history.

## Data Availability

Whole genome sequence data generated in the course of this study is available from the European Nucleotide Archive under study accession number PRJEB33877 [66]. Custom scripts are available at the github repository https://github.com/szutsgroup/hrmutants [67]. Identifiers of the analyzed TCGA samples are listed in Additional file 1: Table S10. Mutation annotation files that formed the basis of the TCGA analysis can be downloaded from the Broad GDAC Firehose portal using instructions provided by Riaz et al. [40].
