# Supplementary material for: Correlation of homologous recombination deficiency induced mutational signatures with sensitivity to PARP inhibitors and cytotoxic agents
Source: Genome Biol. 2019 Nov 14;20:240. doi: 10.1186/s13059-019-1867-0 (PMC6857305; doi:10.1186/s13059-019-1867-0)
Supplement: Supplementary file 2 — Additional file 2: Figure S1. Shows SNV spectra normalised to the frequency of each triplet occurrence. Figure S2. Shows the NMF output used for defining signatures HRD and BG. Figure S3. Shows the derivation of SNV signatures on a dataset that includes pre-existing mutations in the ancestral clones. Figure S4. Correlates detected SNV spectra and signatures with COSMIC SNV signatures. Figure S5. Shows a simulation of separating ‘featureless’ SNV signatures. Figure S6. Shows a deconstruction of detected indel datasets into indel signatures. Figure S7. Shows the results of amplicon sequencing across Cas9 cut sites. Figure S8. Shows drug sensitivity measurements on ancestral and descendent clones. Figures S9 and S10. show the contribution to the SNV load of TCGA samples of COSMIC v2 and v3 SNV signatures, respectively. [file 13059_2019_1867_MOESM2_ESM.pdf]

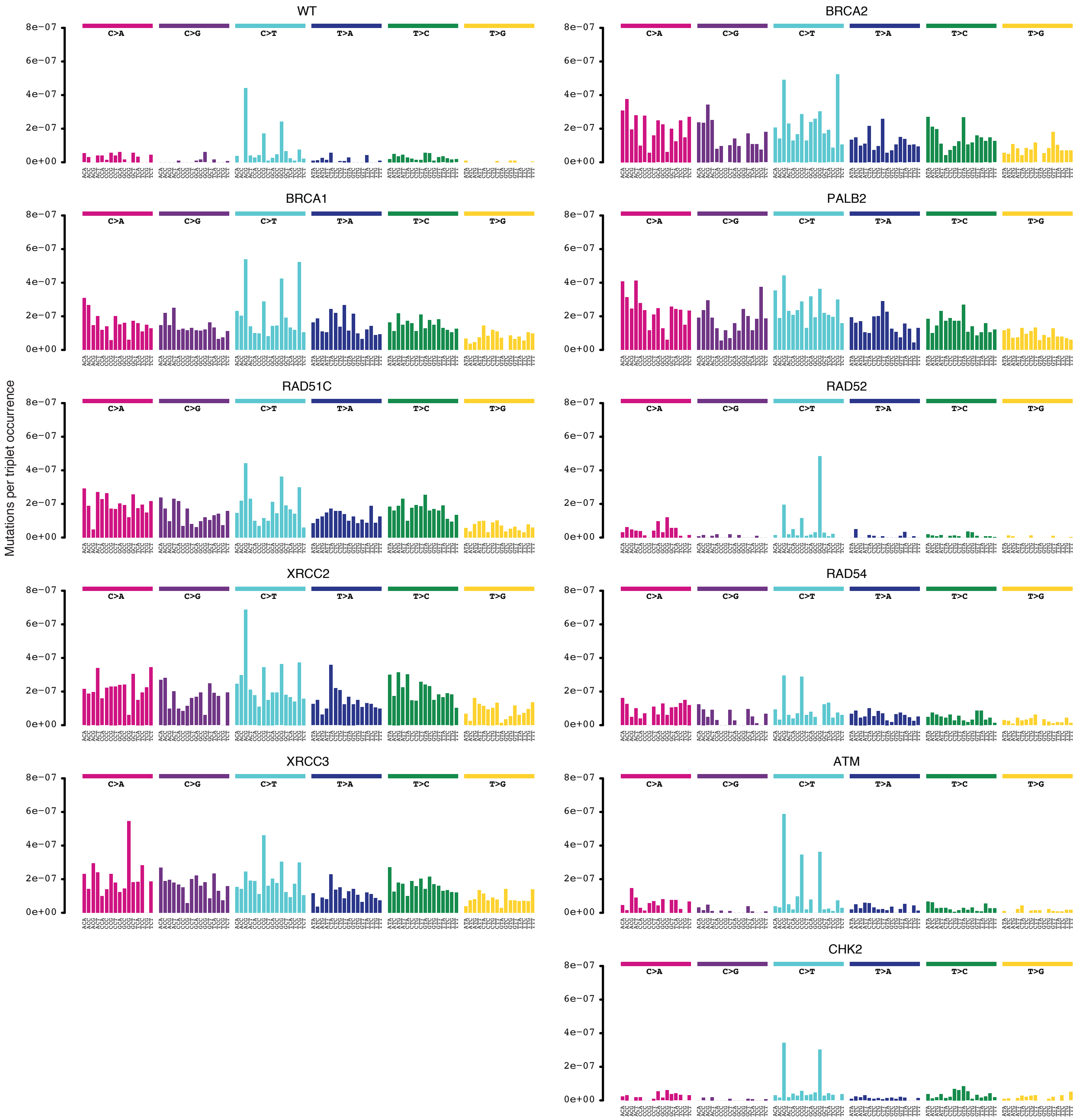

Figure S1.

Triplet spectrum of spontaneous SNVs in wild type (WT) DT40 cells and in the indicated knock-out cell lines, normalised to the frequency of each triplet occurrence in the chicken genome.

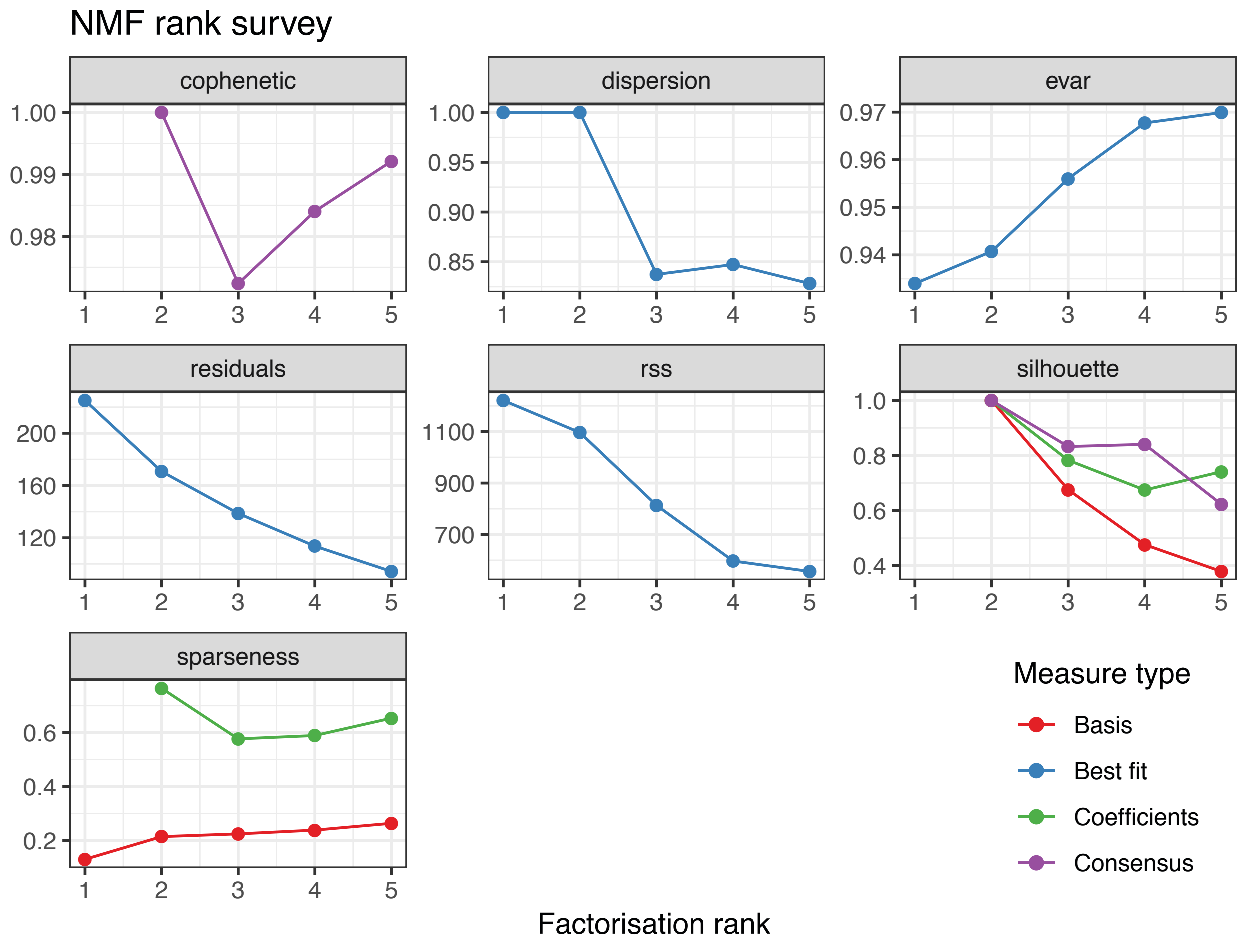

Figure S2.

Non-negative matrix factorisation output for deriving signatures HRD and BG, from the R package *MutationalPatterns*. A factorisation rank of 2 was chosen based on the cophenetic correlation coefficient and the residual sum of squares (rss) values.

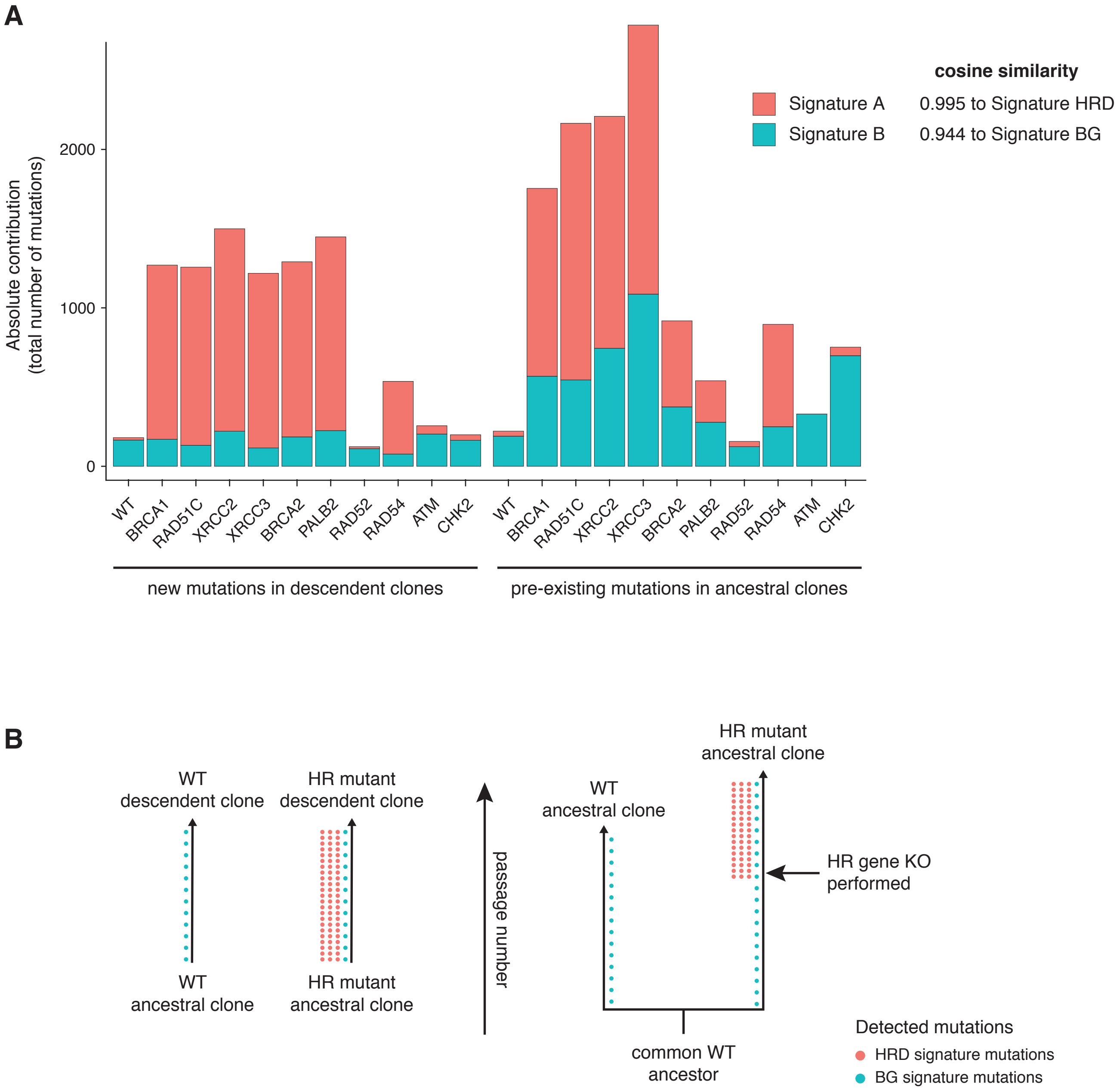

Figure S3.
(A) The derivation of two de novo SNV signatures using non-negative matrix factorisation on a dataset comprising all the new mutations detected in the sequenced descendent clones (summed for each genotype), and all pre-existing mutations in each ancestral clone, which arose since the particular cell lineage separated from its last common ancestor with the other cell lines. The number of detected mutations in the ancestral clones gives an indication about the amount of passaging each lineage has undergone (first as wild type, later as knock-out). The detected signatures show high similarity to signatures HRD and BG defined only on the basis of new mutations in the descendent clones, as shown. (B) A schematic diagram of mutations detected in the experimentally derived descendent clones (left) and in the ancestral clones (right). Only two genotypes are shown for clarity. Pre-existing mutations are detected if they arose since the last common ancestor. As this common ancestor predates the HR gene knockout event, prior to which the HR mutant lineage also had a WT genotype, the HR mutant ancestral clones can contain a variable proportion of HRD and BG signature mutations.

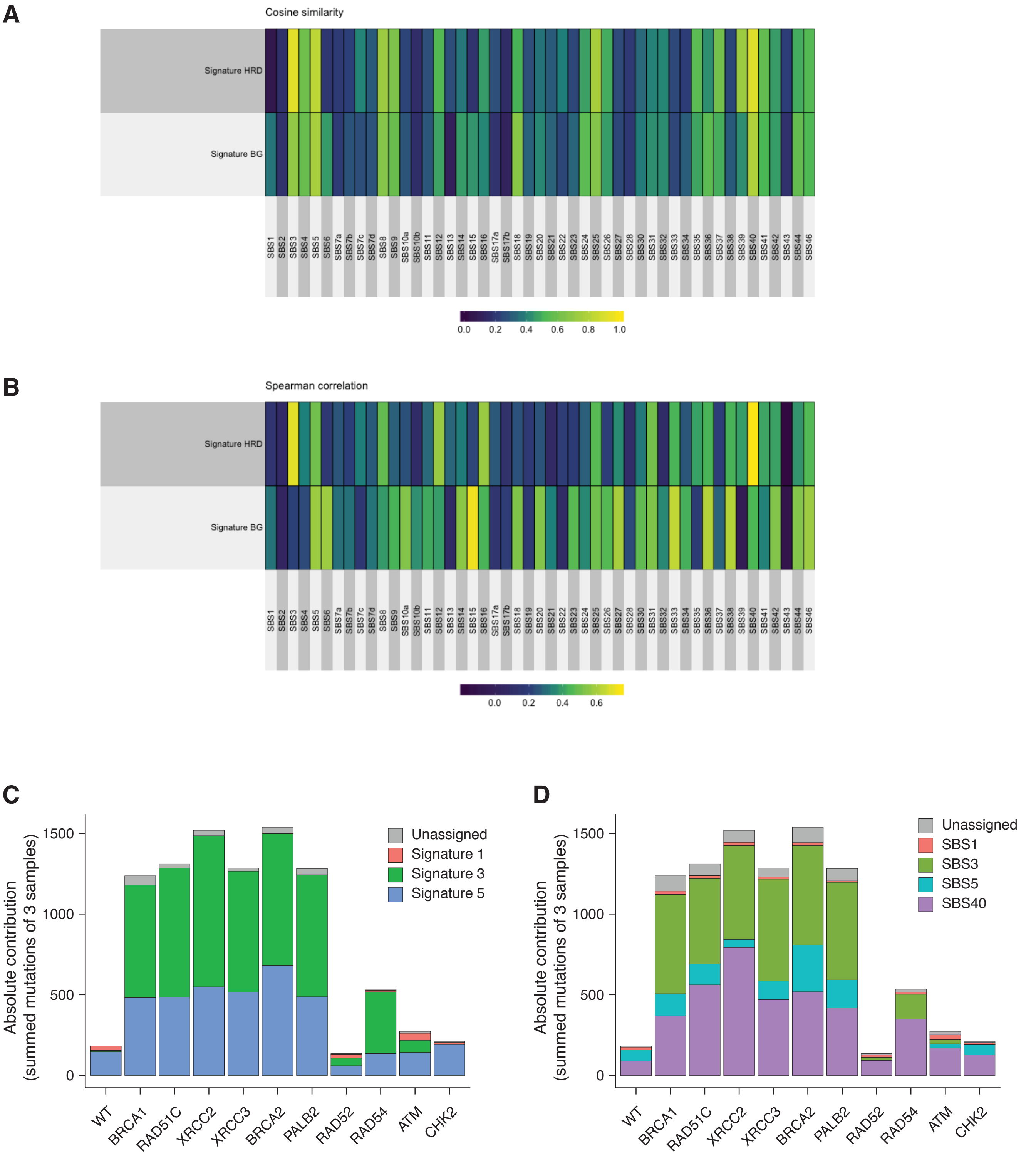

Figure S4.

(A, B) Correlation heat map of the experimentally derived triplet signatures to COSMIC version 3 signatures (Alexandrov et al., 2018, bioRxiv, https://doi.org/10.1101/322859) using cosine similarity (A) or Spearman's rank correlation (B). The experimental signatures were adjusted with the ratio of triplet occurrence frequencies in the human and chicken genomes before comparison.

(C) Deconstruction of the experimentally derived SNV spectra using only COSMIC version 2 signatures 1, 3 and 5. (D) Deconstruction of the experimentally derived SNV spectra using only COSMIC version 3 signatures 1, 3 and 5 and 40.

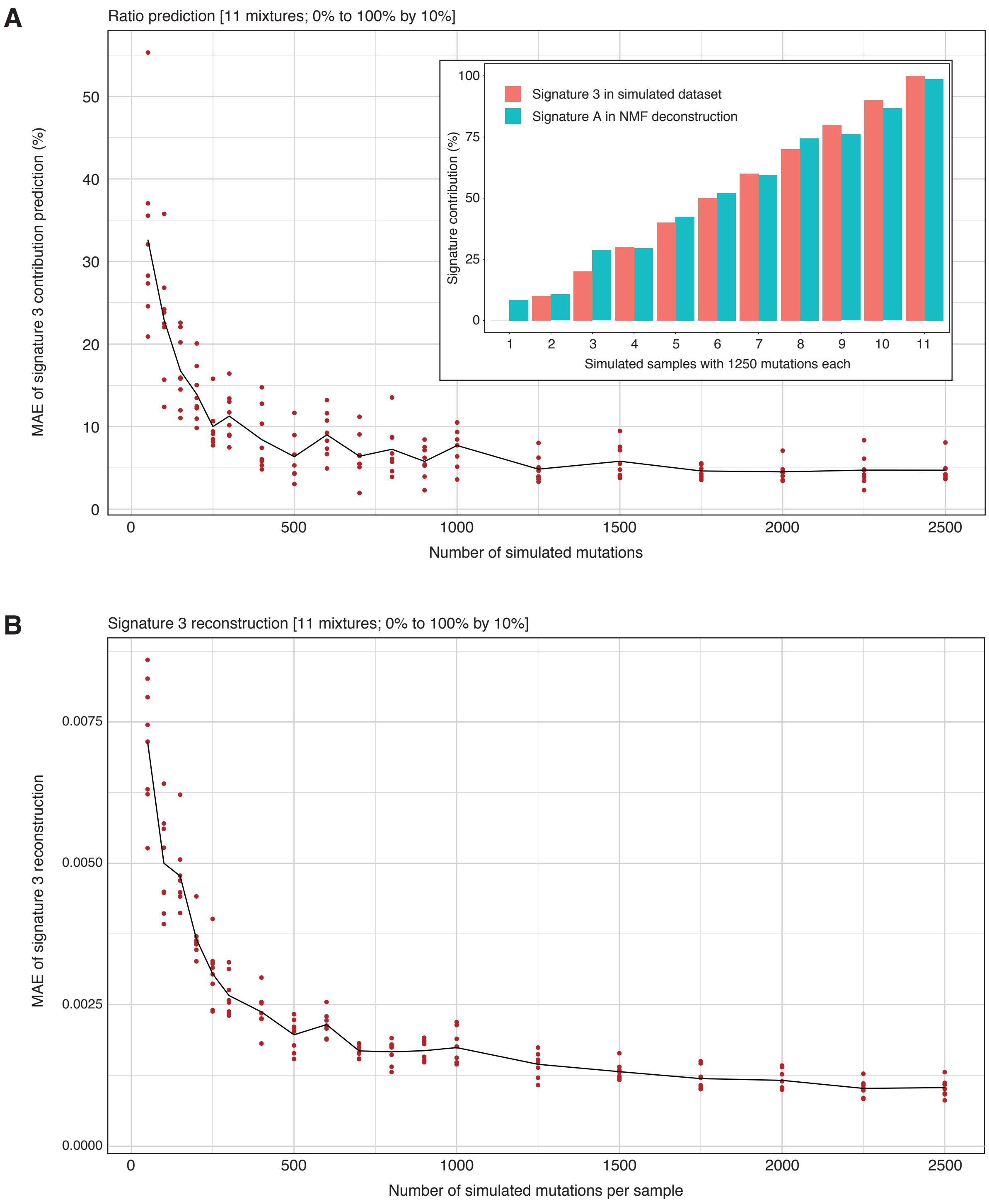

Figure S5.

The accuracy of separating ‘featureless’ SNV signatures.

(A) Simulated datasets of eleven samples were made, each sample containing a different proportion of randomly selected mutations with a COSMIC signature 3 spectrum, in 10% increments (0%, 10%, 20% etc.) as shown on the inset in red. The remaining mutations were drawn with a signature 5 spectrum. Non-negative matrix factorisation was used to separate the datasets into two signature components (A and B), and the mean absolute error (MAE) was calculated between the pre-set signature 3/5 ratio and the derived signature A/B ratio for each dataset. This was performed for different numbers of mutations per sample eight times (red markers). The mean prediction error of the repeats is shown in black. The inset shows a typical result for 1250 mutations per sample, which models the experimental dataset most closely. (B) The success of recreating signature 3 by NMF, calculated as the MAE of comparing signature A to signature 3. Eight repeats are shown (red markers), the mean error of the repeats is shown in black.

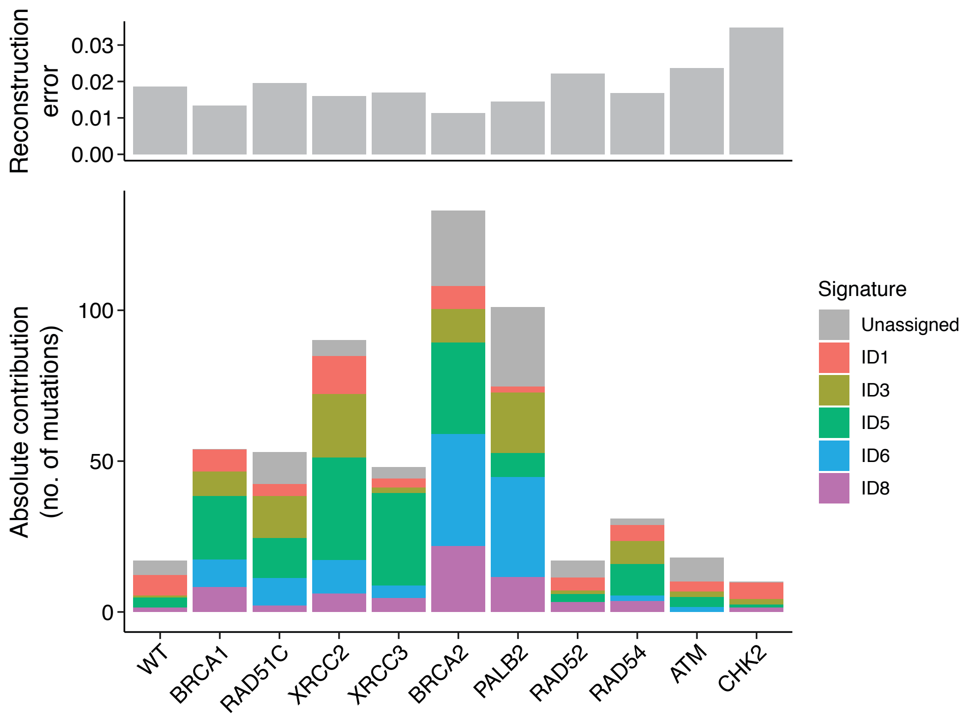

Figure S6.
Deconstruction of short insertion and deletion datasets into indel signatures. Genotype-specific cumulative short indel datasets were deconstructed using the most dominant COSMIC version 3 short indel signatures (Alexandrov et al., 2018, bioRxiv, https://doi.org/10.1101/322859) as indicated. The error (root-mean-squared deviation) of reconstructing the dataset with the detected signatures is shown on the top panel.

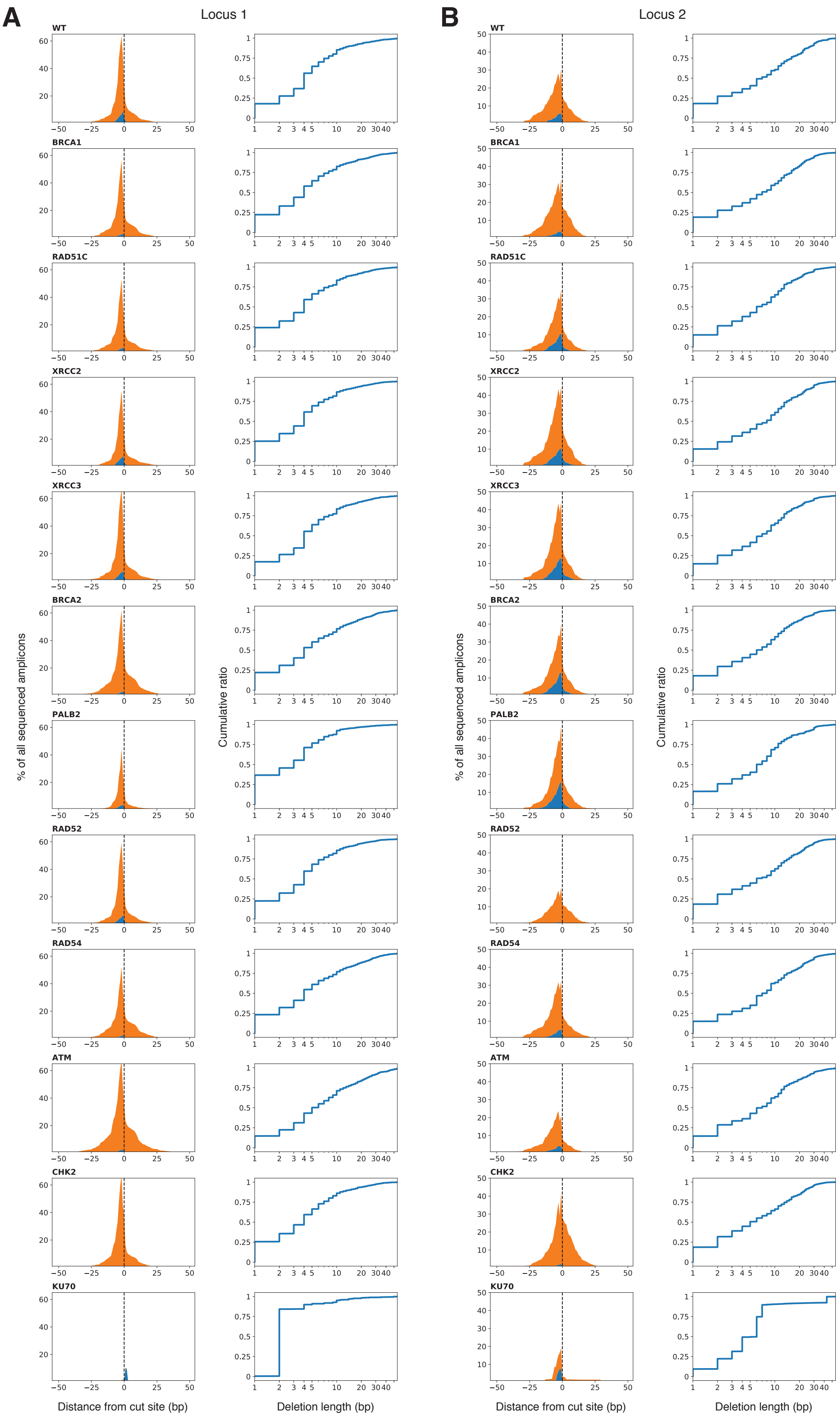

Figure S7.
(A, B) The genetic dependence of deletion formation at CRISPR-induced double strand breaks at two different genomic loci. Left panels, stacked view of pure deletions (orange) and deletions with insertions (blue), shown as the percentage of all sequenced amplicons obtained from genome preparations of cell populations sorted for successful transfection with a plasmid expressing Cas9 and the respective guide RNA. Right panels, cumulative size distribution of deletions.

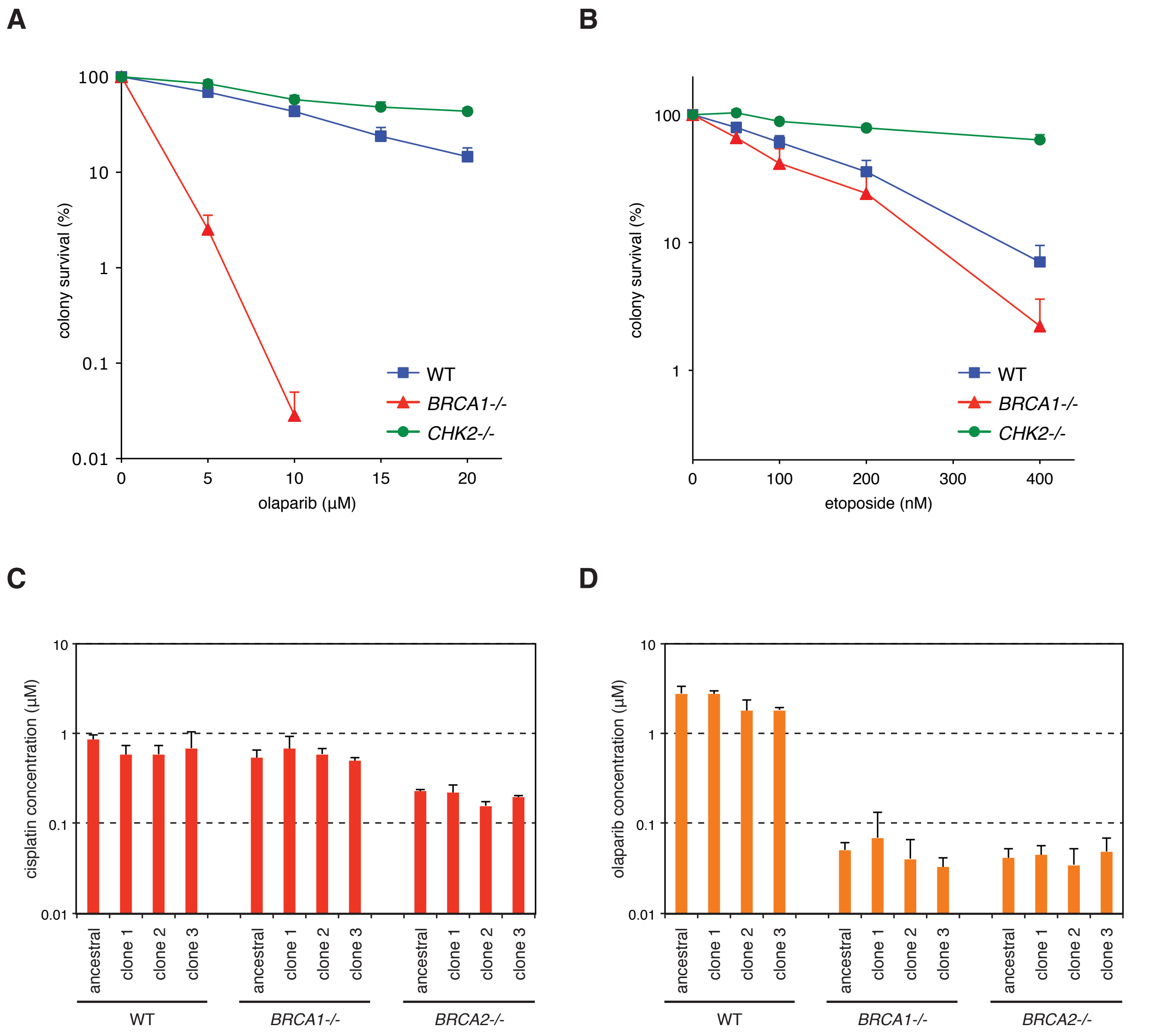

Figure S8.
(A, B) Colony survival assay following the treatment of the indicated cell lines with olaparib (A) or etoposide (B) for 24 hours. The mean survival percentage and S.E.M. of four independent measurements is shown.
(C, D) Results of cytotoxicity measurements showing the mean IC50 value and S.D. of three independent treatments with cisplatin (C) or olaparib (D). The ancestral clone and the three sequenced descendent clones (clone 1, 2, 3) of the indicated cell lines were used.

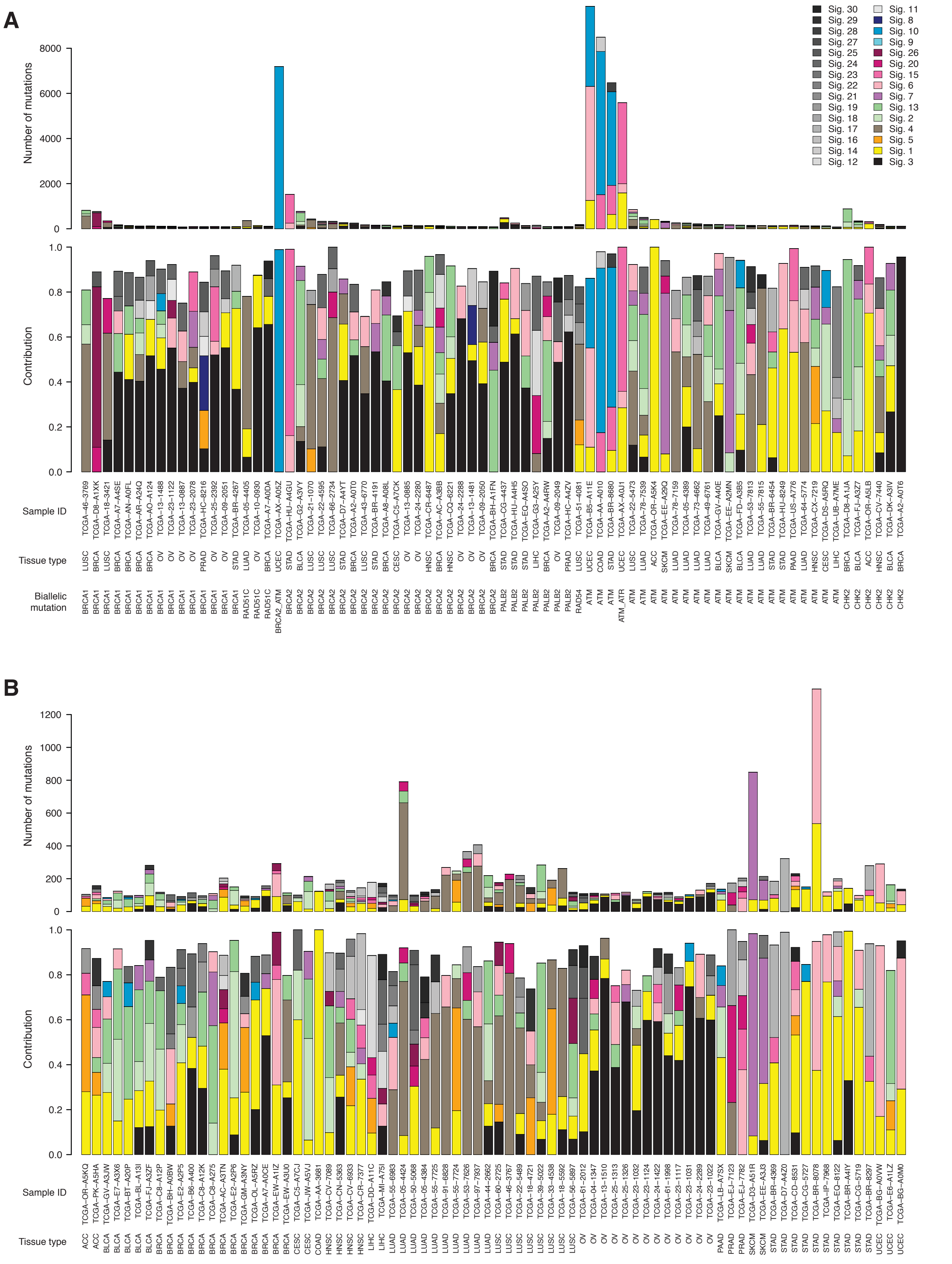

Figure S9.

(A) Contribution of 30 COSMIC SNV signatures to the SNV load of TCGA whole exome tumour samples bearing biallelic inactivating mutations in the indicated genes. (B) The same analysis in a matched random control sample set with no biallelic inactivating mutations in any of the investigated genes. The number of samples of each tissue type (shown below) is equal between (A) and (B).

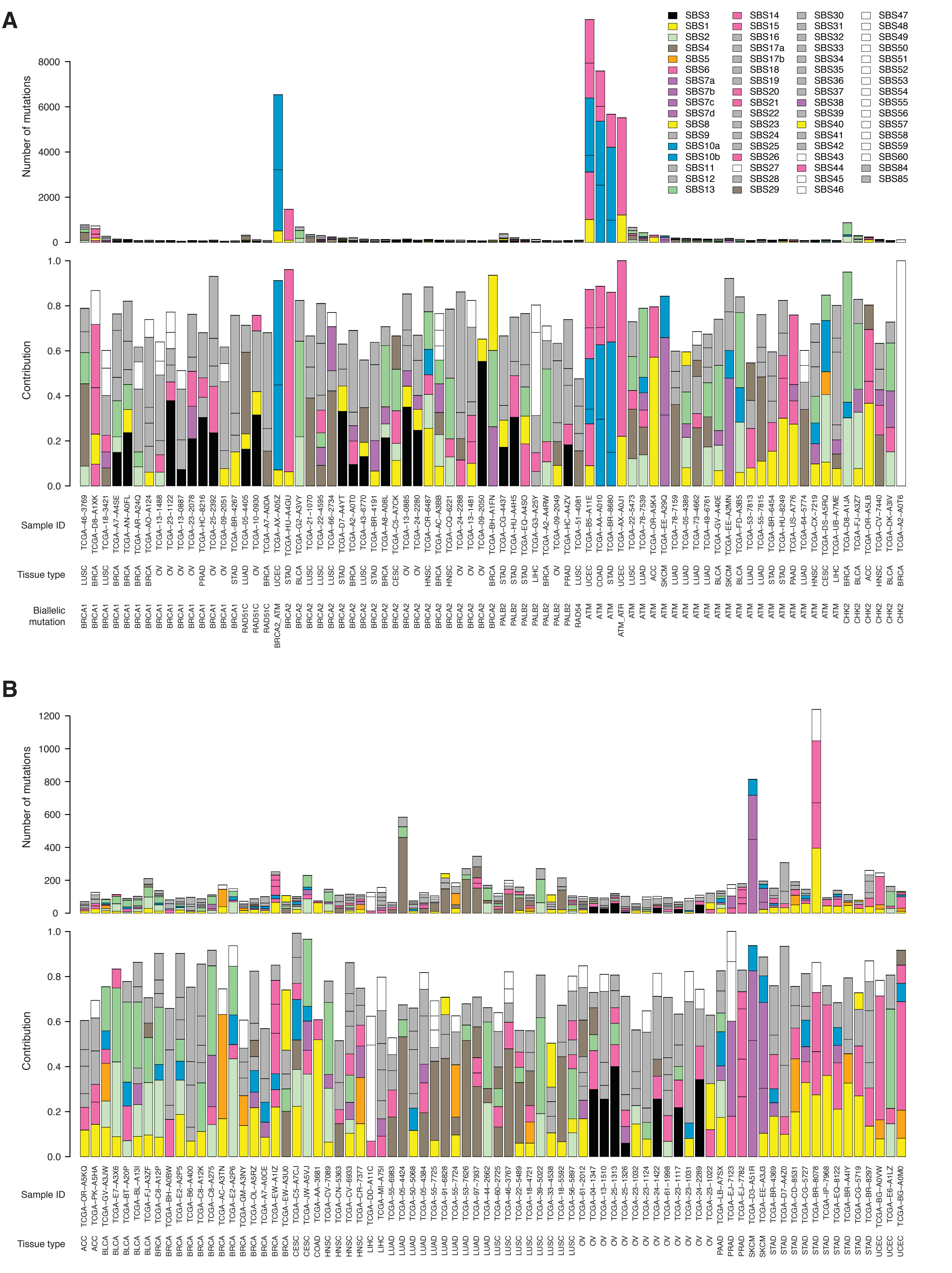

Figure S10.

(A) Contribution of COSMIC version 3 base substitution signatures to the SNV load of TCGA whole exome tumour samples bearing biallelic inactivating mutations in the indicated genes. (B) The same analysis in a matched random control sample set with no biallelic inactivating mutations in any of the investigated genes. The same sample set was analysed as in Figure S9.
