# Supplementary material for: Correlation of homologous recombination deficiency induced mutational signatures with sensitivity to PARP inhibitors and cytotoxic agents
Source: Genome Biol. 2019 Nov 14;20:240. doi: 10.1186/s13059-019-1867-0 (PMC6857305; doi:10.1186/s13059-019-1867-0)
Supplement: Supplementary file 3 — Additional file 3. Review history. [file 13059_2019_1867_MOESM3_ESM.docx]

Review History

**First round of review**

**Reviewer 1**

**Are you able to assess all statistics in the manuscript, including the appropriateness of statistical tests used?**

Yes, and I have assessed the statistics in my report.

**Comments to author:**

The study of Dr. Ádám Póti used whole genome sequencing of an isogenic knock-out cell line panel, a universal HR deficiency specific base substitution was found. The results suggest that no single genomic mutational class shows perfect correlation with sensitivity to common treatments, but the contribution of COSMIC signature 3 to base substitutions, or a combined measure of different features, may be reasonably good at predicting platinum and PARP inhibitor sensitivity. The conclusions adequately supported by the data shown.
It is a great effort added to this area. However, there are still several questions that need to be clarified.

General comments:
1.     For DNA sequencing and mutation calling, the authors used IsoMut to call SNVs and short indels with default settings. I believe the output should have lots of false positive. How the authors to filters these false positive results? The lasted GATK should be applied in the analysis. It is reasonable to me to use the same or similar analysis pipeline as whole exome sequencing of TCGA samples from the Broad GDAC Firehose portal. The same problem is structural variations detection using CREST.

2.     Please elaborate the grouping criterion for Signature HRD and Signature BG and provide some statistical supports.

3.     Why using Cosine similarity to calculate the correlation between 30 COSMIC signatures and Signature HRD and BG. Do other methods work well and COSMIC triplet signature 3 still be the most significant?

4.     Base on the SNV load of TCGA whole exome tumor samples, contribution of 30 COSMIC SNV signatures seem to have specificity in different tumors. Does this work can explain the mechanism of the formation of this signature pattern.

5.     It is better for the authors to make their analysis code public available, eg. using github. 

Minor:
1.     It is better to have some explains for what it is the meaning of COSMIC signature 3. For general readers, they cannot understand COSMIC signature 3.

2.     Add some description of the experimental workflow in Figure 1a

3.     Give the full name of S.E.M. in Figure 1b

4.     Give the explanation of De novo NMF

5.     Give the full names of Signature HRD and Signature BG in Figure 2 legend

**Reviewer 2**

**Are you able to assess all statistics in the manuscript, including the appropriateness of statistical tests used?**

No, I do not feel adequately qualified to assess the statistics.

**Comments to author:**

Review Poti et al. 
Poti et al take a (mostly existing) set of HR-pathway knockout cell lines, generate WGS data and performed mutational signature analysis to assess whether all HR-defects give rise to the same mutational profile, with the overarching aim of assessing whether a one-glove-fits-all test for HR deficiency suffices to predict sensitivity to parp-inhibitors.

Major:
My main concern lies in the conclusions drawn on the SNV spectra. For the most mutagenic knockouts, the authors report ~500 SNVs per clone, of which ~100 are attributes to SBS5, so this leaves only ~400 mutations for SBS3. Being so-called "featureless" signatures, distinguishing SBS3 from SBS5 and SBS40 requires much higher mutation counts (after all, 400 mutations to SBS3 means that that's only 4 mutations on each of the peaks of the 96-channel signature). The effect of noise would be tremendous.
I do not doubt that the authors observed SBS3 in these clones (its aetiology is well established), but my concern is that they report that they observe no differences in SNV spectra, but they do observe differences in deletions.
Below comments/questions/suggestions arise when taking the above into consideration:
-     Can the authors show that the mutation counts that they obtained is sufficient to differentiate if a difference between the different knockout clones would be observed? (i.e. are these mutation counts high enough to give the required resolution)
-     If the answer to the above is no, then the authors need to increase the numbers of mutations, in order to substantiate their conclusions: (and either way, show that the mutation counts would be high enough to differentiate subtle differences in the SNV spectra); Some possibilities would be: 
o     Increase the culture time
o     Comparing the clones to the parental DT40 line instead of the first generation clones. The current experimental setup is excellent to establish that all the different HR knockouts give similar levels of mutagenesis (although I don't think the authors really make this point, it might be worth mentioning…); but from the references I gather that most of these clones have been in culture for a substantial amount of time; comparing the already existing sequencing data to the parental clones would give a must higher count of mutations. (of course there would also be much more SBS1, 5 and 40 present, but I would expect most mutations to still be associated with SBS3, similar to the 60% that is attributed to SBS3 in their current manuscript)

Other Major revisions required:
-     The authors did not take into account the possibility of differences in trinucleotide abundance in chickens compared to humans. All signature assignments should be redone after compensating for possible differences there.
-     The authors have made no mention of the new set of mutational signatures published by the PCAWG consortium; I guess this is a choice, but I would strongly advise the authors to reanalyse using the new signature set instead of the old one; if they chose to stick to the old set of signatures in the manuscript, I expect them to at least put the analysis using the new signature set in the supplementary data.
-     For the indel analysis, the authors seem to be-inventing the wheel, describing microhomology for small deletions. The PCAWG consortium has established an 83-class classification for indels, which includes microhomology classes. The authors need to apply this same classification and compare to the PCAWG results (PCAWG identified indel signatures also, including one that associates with mutational signature 3…; ID6; but this actually has relatively low number of deletions with only 1bp microhomology; the authors should discuss this). I am aware of 2 softwares that are available for generating the indel signatures according to the PCAWG classification (From the Alexandrov lab sigProfilerMatrixGenerator (python framework) and from the Rozen lab ICAMS (R-package)), both are available from github.

Minor:
-     After testing common cytotoxic agents the authors conclude that CHK2 loss doesn't lead to HR deficiency phenotype; the authors should substantiate this by showing that after CHK2 loss HR still works (i.e. that there are backup mechanisms to take over if CHK2 is lost); I suggest γ-H2AX or something of that sort to show this.
-     The authors show increased resistance to platinum agents in CHK2-/- cells, which they validate using colony formation assay; please also look at growth speed, and do similar testing with other compounds in which you also see CHK2 kd leading to resistance (e.g. olaparib + etoposide)
-     The authors report differences in deletion spectra, and not in SNV spectra; is there also a difference in the ratio of deletions vs SNVs?
-     Why was XPC one of the targets of the CRISPR DSB repair experiment? Although XPC is mainly a GG-NER pathway member, there are reports that it also has a role in DSB repair (NHEJ), so I think this choice is quite unfortunate.
-     The authors need to include basic sequencing statistics in the supplementary information (coverage etc)
-     I think the manuscript could improve in terms of readability if the authors would ask a native English speaker to go through it

The following is merely a suggestion, as I think it would improve their manuscript
-     I suggest considering adding a schematic of the HR pathway indicating which proteins are/are not essential as evidenced by their results (and to what cytotoxics they sensitize?)

**Reviewer 3**

**Are you able to assess all statistics in the manuscript, including the appropriateness of statistical tests used?**

Yes, and I have assessed the statistics in my report.

**Comments to author:**

Homologous recombination (HR) repair deficiency arising from defects in BRCA1 or BRCA2 is associated with characteristic patterns of somatic mutations. The aim of this study was to determine which HR or checkpoint gene defects cause somatic mutational processes akin to those seen in BRCA1 or BRCA2 deficient cells, and whether the presence of mutational signatures correlates with treatment sensitivity. Whole genome sequencing (WGS) was performed on isogenic chicken knock-out cell lines, each of which harbors homozygous disruptions of one of eight key HR genes plus ATM and CHK2. SNVs, indel and large structural rearrangements were called from the WGS data. Mutational patterns and drug efficacies were compared among these isogenic knock-out cell lines. The authors concluded that the inactivation of ATM and CHK2 has negligible mutagenic consequences. In addition, SNV mutagenesis showed good but imperfect correlation with PARP inhibitor sensitivity, while the mutagenic processes were poor predictors of sensitivity to a range of common cancer cytotoxics. However, I have several major concerns on this study:
1. The cell lines from various sources with different number of passages were used in the study. A growing body of literature has demonstrated passage number affects a cell line's characteristics over time. Cell lines at high passage numbers experience alterations in morphology, response to stimuli, and growth rates, compared to lower passage cells. Abundant somatic mutations observed in these cell lines may result from passage-dependent accumulation especially during DNA replication and HR deficiency stress. The newly established cell line RAD54-/- in this study had much lower mutation rate than the previously established cell lines. Did this observation reflect the passage-dependent accumulation of somatic mutation? It is essential to rule out the potential confounding effects of passage-dependent accumulation on the results and conclusions made in this study. It would be interesting if the authors re-establish BRCA1 or BRCA2 KO cell lines in a similar way to RAD54-/-  and examine the mutational similarity between the new established cell lines and previous ones. 
2. These cell lines were performed for WGS at 10X coverage (30GB sequencing data). Is this depth of sequencing coverage sufficient to accurately detect indels and large structural rearrangements? Sanger sequencing is required to validate the accuracy of variant calling pipeline in these WGS samples. 
3. As indicated above, the passage-dependent confounding effects should be ruled out in the cytotoxicity assays. 
4. To understand whether the different deletion phenotypes are caused by the influence of a partially disrupted HR pathway on DNA double-strand break (DSB) repair, the authors investigated the spectrum of mutation events at CRISPR/Cas9-induced blunt ended DSBs at two different genomic loci using amplicon sequencing. They observed that CRISPR/Cas9-induced deletions are quite different from spontaneous deletions resulted from HR gene defects and thus concluded "Spontaneously arising short deletions are not the consequence of double-strand break repair". This is not true as the DSB repair that produces spontaneous deletions is not identical to CRISPR/Cas9-induced blunt ended DSB in some circumstances.

**Authors Response**

**Point-by-point responses to the reviewers’ comments:**

Comment

*Response*

Reviewer reports: 

Reviewer #1: The study of Dr. Ádám Póti used whole genome sequencing of an isogenic knock-out cell line panel, a universal HR deficiency specific base substitution was found. The results suggest that no single genomic mutational class shows perfect correlation with sensitivity to common treatments, but the contribution of COSMIC signature 3 to base substitutions, or a combined measure of different features, may be reasonably good at predicting platinum and PARP inhibitor sensitivity. The conclusions adequately supported by the data shown. 
It is a great effort added to this area. However, there are still several questions that need to be clarified. 

General comments: 
1. For DNA sequencing and mutation calling, the authors used IsoMut to call SNVs and short indels with default settings. I believe the output should have lots of false positive. How the authors to filters these false positive results? The lasted GATK should be applied in the analysis. It is reasonable to me to use the same or similar analysis pipeline as whole exome sequencing of TCGA samples from the Broad GDAC Firehose portal. The same problem is structural variations detection using CREST.

*We agree that false positives are an important issue in mutation detection, and we purpose-made the IsoMut tool with this in mind for analysing datasets of isogenic samples. In our paper describing IsoMut (Pipek et al, 2017; cited in the manuscript) we demonstrated that it is a tuneable tool where the rate of false positives can be set to any required level using internal controls. IsoMut works by detecting unique mutations in a single sample amongst a simultaneously analysed sample set. We sequenced and included the ancestral clones in the analysis precisely to provide internal controls for false positives, as any unique mutation found in these clones must be a false positive (otherwise it would also be present in the descendent clones). To make this clearer in the manuscript, we added a sentence in the methods “The output was post-filtered such that no more than five SNVs and one indel were detected in the ancestral clones, as mutations detected as unique in these samples provide an internal control for false positives.“ We also added the mutation numbers detected in the ancestral clones to Supplementary table S4, their original omission was an oversight. 
In our IsoMut paper we made comparisons with GATK’s Mutect and Mutect2. We found that we could optimise these using a ‘panel of normals’ for a similarly accurate detection to what we achieved with IsoMut, but this required an up to 170-fold longer run time. Therefore we do not see a reason to use Mutect2 for mutation detection in this type of isogenic genomic dataset. 
Having said that, we did use the IndelRealigner tool from the GATK toolkit. Our pipeline is described in Zamborszky et al (2017) Oncogene, and referenced in the methods section of this manuscript. 

It is true that the CREST output contains many false positive structural variations. To deal with this issue, we introduced post-flitering steps that are well described in the methods: “The post-filtering of the CREST output utilised three filters to deem a structural event valid: both breakpoints had to be unique among the samples, both breakpoints had to be covered by reads in all of the samples and the structural variant had to be supported by at least five soft clipped reads.“ CREST works on ancestral-descendent sample pairs, the ancestral samples therefore cannot provide a negative internal control. However, the fact that we detected zero rearrangement events in several samples, most notably in most WT, ATM and CHK2 samples, argues against the detection of false positives. We were also able to check most detected SV events in IGV (integrated genome viewer) to confirm their validity.* 


2. Please elaborate the grouping criterion for Signature HRD and Signature BG and provide some statistical supports. 

*We made clearer in the revised manuscript that the NMF procedure was performed on the 11 cumulative triplet mutation datasets. It is important to note that NMF is a simple unsupervised mathematical method. The only influence we had on the outcome was the choice of the number of signatures (the factorisation rank). This is done based on the detailed output of the NMF software tool, basically the recommended choice is the highest factorisation rank before the cophenetic correlation coefficient and the rss value drops off. This is already described in the methods “De novo NMF decomposition and fitting of triplet signatures was performed using the R package MutationalPatterns (Blokzijl et al., 2018). During de novo NMF, an optimal component number of two was chosen based on the cophenetic correlation coefficient and the residual sum of squares values (Fig. S2).” Further information is available in the cited paper. 
To clarify that NMF was an unsupervised learning approach, and the biological correlations were only made on its results, we added a sentence to the results section: “The two signatures resulting from this unsupervised approach appeared to make good biological sense.“* 

3. Why using Cosine similarity to calculate the correlation between 30 COSMIC signatures and Signature HRD and BG. Do other methods work well and COSMIC triplet signature 3 still be the most significant? 

*As cosine similarity is primarily used for comparisons in high-dimensional positive spaces, it is in our opinion particularly suitable for comparing 96-category SNV signatures. However, in addition to cosine similarity we also tested the correlations by calculating Spearman’s rank correlation coefficients, a robust test that is insensitive to the distribution of the data. Both tests showed greatest correlation of Signature HRD with COSMIC signature 3 as shown on Figure 2c and 2d. The correlation values are given in Supplementary table S5. We re-calculated these values after adjusting the experimental mutation spectra for the human/chicken triplet frequency differences, as asked for by the second referee, and replaced Figure 2 panels c and d. There was no noticeable change in the result.* 

4. Base on the SNV load of TCGA whole exome tumor samples, contribution of 30 COSMIC SNV signatures seem to have specificity in different tumors. Does this work can explain the mechanism of the formation of this signature pattern. 

*Indeed, the contribution of SNV signatures to tumour mutation spectra is very informative about the aetiology of the cancer, demonstrating both the effect of external mutagens (e.g. UV radiation, smoking) and certain tumour driver gene defects (most notably mismatch repair defects, which also correlate with mutational load). This has been well described in a number of publications starting with the seminal Alexandrov et al. (2013) Nature paper, cited in our manuscript. Our analysis was not designed with the aim of making new observations about signatures apart from signature 3, partly due to the restriction of the control dataset to match the sample set with inactivating mutations in the investigated genes. 
Nevertheless, it is interesting that we found a contribution of signature 3 to most ovarian cancer samples without inactivating mutations in the investigated HR genes, and we now point out in the manuscript that this suggests HR defects in these tumours: “The median contribution of signature 3 to a matched random TCGA sample set with no inactivating mutation in the genes investigated in this study was zero, but there was a considerable number of mostly ovarian cancer samples which nevertheless contained signature 3 mutations, suggesting HR defects“* 

5. It is better for the authors to make their analysis code public available, eg. using github. 

*We have now uploaded the code for the IsoMut mutation detection analysis and the de novo NMF (R scripts) and the classification of CRISPR-induced mutations (python script) to https://github.com/szutsgroup/hrmutants, and noted this in the methods section. All other analyses used publicly available tools, which we referenced.* 

Minor: 
1. It is better to have some explains for what it is the meaning of COSMIC signature 3. For general readers, they cannot understand COSMIC signature 3. 

*We have added a sentence to the introduction “COSMIC signature 3 is one of a set of somatic base substitution signatures identified in cancer genomes (Alexandrov et al., 2013; COSMIC, 2019)” and referenced the COSMIC mutational signatures web page in addition to the first publication that described these signatures.* 

2. Add some description of the experimental workflow in Figure 1a 

*We have added the following explanation to the figure legend: “The experiments were started with an ancestral single cell clone. After 50 days of culturing, a further cloning step was performed. Genomic DNA was extracted from each ancestral and descendent clone as soon as a sufficient number of cells was available.”* 

3. Give the full name of S.E.M. in Figure 1b 

*We have added the full name to the figure legend.* 

4. Give the explanation of De novo NMF 

*Please see the reply to comment 2 above.* 

5. Give the full names of Signature HRD and Signature BG in Figure 2 legend 

*This is a journal style issue (the abbreviations are already resolved in the main text) but we have added them to the figure legend as well.* 


Reviewer #2: Review Poti et al. 
Poti et al take a (mostly existing) set of HR-pathway knockout cell lines, generate WGS data and performed mutational signature analysis to assess whether all HR-defects give rise to the same mutational profile, with the overarching aim of assessing whether a one-glove-fits-all test for HR deficiency suffices to predict sensitivity to parp-inhibitors. 

Major: 
My main concern lies in the conclusions drawn on the SNV spectra. For the most mutagenic knockouts, the authors report ~500 SNVs per clone, of which ~100 are attributes to SBS5, so this leaves only ~400 mutations for SBS3. Being so-called "featureless" signatures, distinguishing SBS3 from SBS5 and SBS40 requires much higher mutation counts (after all, 400 mutations to SBS3 means that that's only 4 mutations on each of the peaks of the 96-channel signature). The effect of noise would be tremendous. 
I do not doubt that the authors observed SBS3 in these clones (its aetiology is well established), but my concern is that they report that they observe no differences in SNV spectra, but they do observe differences in deletions. 
Below comments/questions/suggestions arise when taking the above into consideration: 
- Can the authors show that the mutation counts that they obtained is sufficient to differentiate if a difference between the different knockout clones would be observed? (i.e. are these mutation counts high enough to give the required resolution) 

*The y axis of the relevant figures (Figure 2a, old S3 - now S4c) was slightly misleadingly labelled, with the ‘number of mutations’ scale running to 500 mutations, suggesting that only up to 500 mutations were used for the de novo signature extraction. Instead, while the graph shows the mean number of mutation per clone of the indicated phenotype, the NMF procedure used the sum of all mutations per genotype. With 3 clones sequenced per genotype, this amounts to 1200-1500 mutations in case of the HR deficient clones. We have clarified the axis titles, and made it clear in the Results section that NMF was performed “on the 11 cumulative triplet mutation datasets”. 
To address the question whether this number of mutations is sufficient to separate different proportions of signature 3 and 5, we generated simulated mutation datasets. In each dataset, there were 11 samples (as in the real SNV dataset), with each sample containing a pre-set proportion of randomly selected mutations with a signature 3 spectrum (0, 10%, 20% etc), and the remainder with a signature 5 spectrum. We then performed NMF into two signatures on such simulated datasets with different total mutation numbers per sample. As shown on the new Figure S5A, the mean absolute error between the signature composition of the deconstructed samples and the pre-set proportions was well below 10% at 500 mutations per sample, and did not substantially decrease beyond 1250 mutations per sample. This demonstrates that our mutation sets were sufficiently large to distinguish between signatures 3 and 5 as well as it is possible to do so. The new Figure S5B also shows that at over 1000 mutations per sample, the accuracy of recreating the mixed signatures by NMF only increases very gradually. 
In addition to the new supplementary figure 5s, we added the following sentence to the main text: 
“Modelling the NMF process on different sized sample sets drawn from pre-set mixtures of signatures 3 and 5 showed that the number of mutations detected in the HR deficient cell lines allows the separation of these signatures with only 5% mean absolute error (Figure S5, Additional File 1), confirming that SNV mutagenesis in the BRCA1, BRCA2, PALB2 and the RAD51 paralog mutants is indistinguishable both in rate and spectrum.”* 

- If the answer to the above is no, then the authors need to increase the numbers of mutations, in order to substantiate their conclusions: (and either way, show that the mutation counts would be high enough to differentiate subtle differences in the SNV spectra); Some possibilities would be: 
o Increase the culture time  *In the light of the above calculations, this was thankfully not necessary.* 

o Comparing the clones to the parental DT40 line instead of the first generation clones. The current experimental setup is excellent to establish that all the different HR knockouts give similar levels of mutagenesis (although I don't think the authors really make this point, it might be worth mentioning…); but from the references I gather that most of these clones have been in culture for a substantial amount of time; comparing the already existing sequencing data to the parental clones would give a must higher count of mutations. (of course there would also be much more SBS1, 5 and 40 present, but I would expect most mutations to still be associated with SBS3, similar to the 60% that is attributed to SBS3 in their current manuscript) 

*We wish to thank the reviewer for the idea of looking into pre-existing mutations, which also helps answering reviewer 3’s major concern about passage numbers. 
As the cell lines were made at different times in different laboratories, we have no access to the parental clones in which the knockouts were made. Also, in most cases the parental wild type cell line would not have been cloned on purpose, therefore such a parental clone was never available. Instead, we were able to look for cell line specific mutations in each of the ancestral clones isolated and sequenced for this study by identifying unique mutations in each ancestral clone when compared to all other sequenced whole genomes of all other genotypes. This was simple to do: we re-ran the IsoMut mutation detection 11 times, each time only including the ancestral clone from one of the genotypes, but all ancestral and descendent clones from all other genotypes so as not to change the total sample number significantly as compared to the original IsoMut run which included all samples. The identified unique mutations are those that are not present in any other cell line, not even in the wild type, i.e. those mutations that arose since the last common ancestor between the analysed cell line and any of the others. In the lineage of descent initially each cell line was wild type, and at some point the gene knock-out was made, and the mutations reflect the sum of this entire period. 
Next, we repeated the NMF as in Fig. 2a with the inclusion of the mutations found in the ancestral clones, roughly doubling the number of mutations in the analysis (the result is shown in Fig. S3). The result was very similar to the original attempt: a signature found in the HR mutants was essentially identical (cosine similarity 0.995). 
It is interesting to note that although the same seven cell lines show pre-existing HRD mutations, the proportion of pre-existing background mutations is higher than the proportion of new background mutations in the descendent clones. This is entirely expected due to the partly wild type lineage, as explained above. 

The above results were added to the methods and results sections, and a new supplementary figure (S3) is also included.* 


Other Major revisions required: 
- The authors did not take into account the possibility of differences in trinucleotide abundance in chickens compared to humans. All signature assignments should be redone after compensating for possible differences there. 

*Indeed we forgot to adjust for the difference in trinucleotide frequencies. We have now re-made Figure 2c and 2d with human-adjusted spectra, and also used such spectra for new comparisons to the version 3 SBS signatures. 
As the differences are very small (as shown graphically in one of our recent papers), there are no noticeable changes in the figures. We inserted a sentence in the methods: “For comparisons to COSMIC triplet signatures, DT40 triplet signatures were adjusted by multiplying with the ratio of triplet occurrences in the human and chicken genomes (Szikriszt et al., 2016).”* 

- The authors have made no mention of the new set of mutational signatures published by the PCAWG consortium; I guess this is a choice, but I would strongly advise the authors to reanalyse using the new signature set instead of the old one; if they chose to stick to the old set of signatures in the manuscript, I expect them to at least put the analysis using the new signature set in the supplementary data. 

*We are aware of the new mutation signatures, and had two reasons against using them. 
The first is their publication status. They are described in a preprint (Alexandrov 2018, BioRxiv), and we have been awaiting a final published form of this study. They are also published on the COSMIC website, but it is not ideal to reference websites as web addresses can change: for example, the COSMIC website reference that we used in our recent papers now points to a different (v3) version of the page, not to the version 2 signatures which were used in those papers. 
The second reason is that we did not find the use of the new signatures very necessary. The v3 signatures quite successfully reproduced the v2 signatures (bar one) and added a number of extra ones. Many of the extra signatures result from external mutagen exposure, which is not relevant to our study of spontaneous mutagenesis, and the remainder mostly describe rare or minor processes. Signature deconstruction performs less reliably the more signatures are used (see below), we therefore decided for simplicity to use the 30-signature v2 set for the deconstruction of tumour WGS mutation data for Figs. 6 and S8. The alternative solution of course is the use of a judiciously selected subset of signatures, optimised for the detection of a certain biological property. However, making such a selection from the v3 signatures is beyond the scope of this study, and it would need many more non-BRCA HR mutant samples. 
Taking the reviewer’s advice on board, we repeated our analyses with v3 signatures, and now provide the following in the supplementary data: 
1. cosine and Spearman similarity comparisons between v3 signatures and the HRD and BG signatures as in fig. 2c, d (see the new figure S4a,b) 
2. a deconstruction to the new SBS 1, 3, 5 and 40 (see the new figure S4d) 
3. A deconstruction of the human WGS samples to all v3 signatures is shown in Fig. S9, showing the contribution of SBS3. We also remarked in the main text that “Using the entire signature set from COSMIC version 3 resulted in lower sensitivity but higher specificity of detecting signature SBS3 in HR mutant samples (Supplementary Fig. S9, Additional File 1).“* 

- For the indel analysis, the authors seem to be-inventing the wheel, describing microhomology for small deletions. The PCAWG consortium has established an 83-class classification for indels, which includes microhomology classes. The authors need to apply this same classification and compare to the PCAWG results (PCAWG identified indel signatures also, including one that associates with mutational signature 3…; ID6; but this actually has relatively low number of deletions with only 1bp microhomology; the authors should discuss this). I am aware of 2 softwares that are available for generating the indel signatures according to the PCAWG classification (From the Alexandrov lab sigProfilerMatrixGenerator (python framework) and from the Rozen lab ICAMS (R-package)), both are available from github. 
 *We opted for a simplified indel classification due to the following considerations: 
(1) The experimental short indel datasets are quite small, with as few as 20 indels per genotype. For COSMIC indel signatures we would need to classify these into 83 classes, which will clearly be variable. Instead, we concentrated on deletions (which were more numerous than insertions, and showed significant changes) and classified them into three simple distinct classes as also done in our recent papers. 
(2) Deconstructing small datasets into many signatures (there are 17 indel signatures in the COSMIC v3 set) is again problematic, and there is an arbitrariness in choosing subsets of signatures for deconstruction. 

Nevertheless, we have now assigned the indels into the 83 categories, and attempted deconstruction. We got the best result with using five ID signatures: 1, 3, 5, 6, 8. Little meaningful deconstruction is seen with the wild type-like cell lines, but the HR mutants appear to fall into two categories, those with a high contribution of ID5 (T deletions from homopolymers, BRCA1, RAD54 and RAD51 paralogs) and those with also a high contribution of ID6 and ID8 (longer deletions with microhomology, BRCA2 and PALB2). However, the reconstruction error is high for all mutants due to the small dataset sizes, and there is a high proportion of unassigned indels. Please look at a figure here: https://www.dropbox.com/s/0uyd75b7vljqyvp/indel_signatures_reconstruction.pdf?dl=0 
We could include such a figure in the manuscript, but we are wary of overinterpreting the data, and we already made the point about increased very short deletions in all HR mutants, plus deletions with microhomology in BRCA2 and PALB2.* 

Minor: 
- After testing common cytotoxic agents the authors conclude that CHK2 loss doesn't lead to HR deficiency phenotype; the authors should substantiate this by showing that after CHK2 loss HR still works (i.e. that there are backup mechanisms to take over if CHK2 is lost); I suggest γ-H2AX or something of that sort to show this. 

*Our conclusion was merely a description of the observed phenotypes in the paper’s dataset: loss of CHK2 does not cause an HR-like SNV phenotype, an HR-like deletion phenotype, and sensitivity to platinum and PARP inhibitors. The suggested experiment would be complex to interpret, as ATM, ATR, CHK1 and CHK2 form a complex network in the cell which is involved in H2AX phosphorylation, therefore H2AX phosphorylation could not be used as an independent measure of HR activity in the CHK2 mutant. The role of CHK2-mediated DNA damage checkpoint in DNA repair has been investigated, and in many settings CHK2 inhibition indeed does not impair DSB repair. We now cite two such reports in the discussion: “This is in agreement with reports showing no impairment of HR repair upon CHK2 inactivation (Huang et al., 2008; Lima et al., 2016).” In addition, please note that our results were obtained in a more physiological setting compared to most previous studies, i.e. we are observing the role of proteins in the response to spontaneously occurring events, not to induced DSBs. Hence the careful phrasing of the conclusion: “the main function of this DNA damage checkpoint mechanism is not the facilitation of correct repair, at least in cells with a moderate amount of endogenous DNA damage. “* 

- The authors show increased resistance to platinum agents in CHK2-/- cells, which they validate using colony formation assay; please also look at growth speed, and do similar testing with other compounds in which you also see CHK2 kd leading to resistance (e.g. olaparib + etoposide) 

*We used short treatments for the colony formation assay, e.g. 1 hour for cisplatin. After the treatment, the cells are plated in the absence of the drug, and we wait until countable colonies appear. This way, colony formation assays measure sensitivity independently of growth rate: all surviving and proliferating cells are counted. With this assay, the growth rate cannot be taken into account. Following the referee’s suggestion, we performed the assay also with olaparib and etoposide, and the assays confirmed the resistance of the CHK2 mutant to these agents. The new results are shown on Figure S7, and cited in the main text.* 

- The authors report differences in deletion spectra, and not in SNV spectra; is there also a difference in the ratio of deletions vs SNVs? 

*The ratio of deletions to SNVs may indeed be a useful classifier amongst HR mutants. In our experiments, six HR mutants showed identical SNV rates (which we now better emphasise, following the referee’s suggestion), therefore this ratio corresponds to the rates of deletion formation, with BRCA2 the highest. 
In tumour data such a measure would be especially useful, as the absolute mutation numbers differ. We attempted to calculate this ratio from the TCGA data we analysed. However, the whole exome data contains too few deletions (Fig. 6b) to make this meaningful. In our opinion, the ratio of microhomology deletions to all SNVs in whole cancer genomes would be a useful separator of BRCA1-like and BRCA2-like HR phenotypes, but the available datasets did not allow us to draw firm conclusions on this.* 

- Why was XPC one of the targets of the CRISPR DSB repair experiment? Although XPC is mainly a GG-NER pathway member, there are reports that it also has a role in DSB repair (NHEJ), so I think this choice is quite unfortunate. 

*XPC was chosen simply because we had an efficient, tested guide RNA for this genomic site in the lab. Whilst we agree that this is an odd choice, we think it does not influence the results for the following reasons: 
(1) A possible XPC knock-out would not immediately affect DNA repair due to the existing mRNA and protein. The time between the Cas9 cut and the repair is likely very short. The time required for DSB repair (mostly by NHEJ), as measured directly using pulsed field gel electrophoresis or comet assays is on the order of 30 minutes (see e.g. Radiat. Res., 148, 285-292) and it is most likely that the pre-existing XPC protein is still available during this time for any repair function. 
(2) The cells were sorted for the presence of the plasmid expressing Cas9 and the guide RNA, and we observed sequence changes in 40-60% of the amplicons covering the target site. This suggests that only one allele was targeted in many, if not most, cells, and the other allele would have been able to continue expressing the XPC protein. 
(3) We repeated the experiment targeting a housekeeping gene, HMBS, and collected very similar results.* 

- The authors need to include basic sequencing statistics in the supplementary information (coverage etc) 

*Sequencing statistics (coverage, insert size, proportion of mapped reads, mapping quality) are now presented in supplementary table S1.* 

- I think the manuscript could improve in terms of readability if the authors would ask a native English speaker to go through it 

*One of the corresponding authors is a native English speaker, we have made small improvements to the text.* 

The following is merely a suggestion, as I think it would improve their manuscript 
- I suggest considering adding a schematic of the HR pathway indicating which proteins are/are not essential as evidenced by their results (and to what cytotoxics they sensitize?) 

*We were contemplating adding an HR pathway scheme, as indeed we usually show these in conference talks. However, we rejected the idea for the manuscript for several reasons: (1) The HR pathway is rather complex, and reviews usually show large figures with many branches of HR: Holiday junction dissolution, HJ resolution, SDSA, SSA etc. (2) Even these large figures mostly show HR as a DSB repair pathway, but HR has other functions that are probably more important to the mutagenic phenotypes: the repair of a broken replication fork, the restart of a collapsed fork, the protection of a stalled fork. (3) The functions of distinct HR factors are not fully known, especially for the RAD51 paralogs, therefore it is not obvious where they should be placed on the pathway schemes. In conclusion, rather than showing a misleading figure it may be better to show no figure at all - but we did include a discussion section on the function of the various HR proteins.* 


Reviewer #3: Homologous recombination (HR) repair deficiency arising from defects in BRCA1 or BRCA2 is associated with characteristic patterns of somatic mutations. The aim of this study was to determine which HR or checkpoint gene defects cause somatic mutational processes akin to those seen in BRCA1 or BRCA2 deficient cells, and whether the presence of mutational signatures correlates with treatment sensitivity. Whole genome sequencing (WGS) was performed on isogenic chicken knock-out cell lines, each of which harbors homozygous disruptions of one of eight key HR genes plus ATM and CHK2. SNVs, indel and large structural rearrangements were called from the WGS data. Mutational patterns and drug efficacies were compared among these isogenic knock-out cell lines. The authors concluded that the inactivation of ATM and CHK2 has negligible mutagenic consequences. In addition, SNV mutagenesis showed good but imperfect correlation with PARP inhibitor sensitivity, while the 
mutagenic processes were poor predictors of sensitivity to a range of common cancer cytotoxics. However, I have several major concerns on this study: 
1. The cell lines from various sources with different number of passages were used in the study. A growing body of literature has demonstrated passage number affects a cell line's characteristics over time. Cell lines at high passage numbers experience alterations in morphology, response to stimuli, and growth rates, compared to lower passage cells. Abundant somatic mutations observed in these cell lines may result from passage-dependent accumulation especially during DNA replication and HR deficiency stress. The newly established cell line RAD54-/- in this study had much lower mutation rate than the previously established cell lines. Did this observation reflect the passage-dependent accumulation of somatic mutation? It is essential to rule out the potential confounding effects of passage-dependent accumulation on the results and conclusions made in this study. It would be interesting if the authors re-establish BRCA1 or BRCA2 KO cell lines in a similar way to RAD54-/- and examine the mutational similarity between the new established cell lines and previous ones. 

*We agree with the reviewer that “somatic mutations observed in these cell lines may result from passage-dependent accumulation” - in fact, given that during the mutagenesis assay the cells are not treated with any external agent, all the mutations we observed can be called passage-dependent. Thanks to the suggestion of reviewer 2, we now know much more about history and number of past passages of each cell line. The analysis presented in the new figure S3 shows pre-existing mutations in the ancestral clones. The majority of pre-existing mutations in all the HR mutants show the HRD spectrum, demonstrating that this analysis indeed found mutations that arose after the cell lines were created. 
We can therefore compare 
- pre existing mutations in ancestral clones, acquired since the split from the last common ancestor 
- mutations accumulated during a 50-day experiment: this current mutation rate is identical for six of the HR mutant lines. 
The results show that the XRCC3 cell line has the highest amount of pre-existing mutations, suggesting that it had been kept in culture the most, followed by XRCC2 and RAD51C, BRCA1, BRCA2 and finally PALB2. There was a six-fold difference in the HRD contribution to pre-existing mutations between XRCC3 and PALB2, yet all six of the mentioned cell lines produced HRD spectrum SNVs at the same rate in our controlled experiments. 
The same phenomenon is apparent with cell lines of the background mutation spectrum. RAD52 was made in our lab, whereas CHK2 was a gift, and was made much earlier. Indeed, CHK2 contained many more pre-existing mutations, but the two lines produced BG spectrum SNVs at the same rate in our controlled experiments. 
In conclusion, this analysis shows that the actual current mutation rates were not influenced by accumulated past mutations. This is the simplest, most logical explanation of the new data. 

To summarise this in the manuscript, we inserted a comment in the main text: “The inclusion in the NMF analysis of pre-existing mutations in the ancestral clones provided a very similar HRD-like signature (cosine similarity 0.995) and showed that the mutation rates do not correlate with past passaging (Supplementary Fig. S3, Additional File 1)“, inserted the new figure S3, and explained the passaging effect in the figure legend: “The number of detected mutations in the ancestral clones gives an indication about the amount of passaging each lineage has undergone (first as wild type, later as knock-out).” 

To provide further information on accumulated mutations during past passages, we listed all the coding mutations in the ancestral clones in a new table S11, cited in the methods section. We found on average 18 coding mutations per cell line, and the number of coding mutations correlated with the total number of pre-existing mutations as shown in figure S3. We can present the following arguments that these mutations do not change the phenotype: 
- all the mutations found were heterozygous, except for a few hemizygous mutations on chromosome Z, and a synonymous homozygous mutation in CHK2 in the CHK2 knockout line (this was likely introduced in the knockout construct) 
- we see no DNA repair genes in the list of affected genes, apart from CHK2, PALB2 and XRCC2 point mutations in the respective knockout lines (likely from the knockout construct, as above) 
- the proportion of synonymous base substitution mutations was 27%, exactly as expected for random mutations with a flat spectrum, arguing against a selectable phenotypic effect. 

As a further comment, we would like to mention that we and the other labs try to keep passage numbers low by limiting the amount of culture time and number of freezing cycles. Indeed, some of the frozen cell stocks thawed for these experiments were 10 years old, frozen at early passages.* 

2. These cell lines were performed for WGS at 10X coverage (30GB sequencing data). Is this depth of sequencing coverage sufficient to accurately detect indels and large structural rearrangements? Sanger sequencing is required to validate the accuracy of variant calling pipeline in these WGS samples. 

*The size of the chicken genome is barely over 1 GB, therefore the average sequencing depth calculated from aligned reads was 27x. This is now presented in Table S1. A coverage of 27x is considered sufficient for mutation detection by all common methods in diploid genomes, and the DT40 cell line genome is almost completely normal diploid, as we published earlier (Molnar et al, 2014. We emphasise this in the revised manuscript: “A mean coverage of 27x was achieved in the near-diploid DT40 genome (Molnár et al., 2014).” 
We published an earlier paper in which we validated the variant calling pipeline (both in terms of false negatives and false positives, as a function of sequencing coverage), please see our reply to point 1 of referee 1. As for rearrangements, please see the same reply about the careful post-filtering we performed to eliminate false positives.* 

3. As indicated above, the passage-dependent confounding effects should be ruled out in the cytotoxicity assays. 

*To address this issue, we thawed frozen stocks of the ancestral clone and descendent clones of three cell lines (WT, BRCA1, BRCA2) and performed cytotoxicity assays on them with two drugs (cisplatin, olaparib). The descendent clones were obtained by simple culturing (i.e. passaging) the ancestral clone, so passage-dependent changes in sensitivity should show up in this measurement. As the new figure S7 shows, the ancestral and descendant clones showed very similar sensitivities, as measured by IC50 values. 
We also performed similar measurements in the past. For example, in our cited paper Zamborszky et al (2017, Oncogene) we compared the MMS sensitivity of ancestral and post-treatment cell DT40 cell clones, which came from a similar 50-day experiment as those in this manuscript, and again observed no difference (Figure 3 in that paper). 
In conclusion, whilst we agree that cell lines can change upon culturing, we do not see evidence of such changes affecting the phenotype of the DT40 lines used in this study. The analysis of coding mutations in the cell lines presented in reply 1 supports this conclusion.* 

4. To understand whether the different deletion phenotypes are caused by the influence of a partially disrupted HR pathway on DNA double-strand break (DSB) repair, the authors investigated the spectrum of mutation events at CRISPR/Cas9-induced blunt ended DSBs at two different genomic loci using amplicon sequencing. They observed that CRISPR/Cas9-induced deletions are quite different from spontaneous deletions resulted from HR gene defects and thus concluded "Spontaneously arising short deletions are not the consequence of double-strand break repair". This is not true as the DSB repair that produces spontaneous deletions is not identical to CRISPR/Cas9-induced blunt ended DSB in some circumstances. 

*We accept the reviewer’s point that the Cas9-induced DSB is not identical to spontaneous DSBs. However, we simply do not know what the range of spontaneous DSBs looks like (what proportion are blunt ended, sticky ended or unligatable due to other accompanying DNA lesions, what proportion occur at replication forks or away from them, what proteins (e.g. topoisomerase II) are bound to what proportion of the DSBs). Therefore all we could do was to use an experimental model that generates a certain type of DSBs. However, the similarity of the BRCA2 and PALB2 spontaneous and Cas9-induced deletion phenotypes, and the reduction of deletions in KU70 mutants suggests that the experiment modelled the outcome of DSBs reasonably well. 

To make our statements more accurate, we changed the title of the relevant results section to make it clear that we concluded about the “very short” deletions that they are not due to DSB repair. We also further emphasised the caveat of using one type of experimental DSB: “The majority of spontaneous deletions observed in HR mutants are thus shorter than those observed at nuclease-induced DSBs. With the caveat that the Cas9 cut does not reproduce all types of potential spontaneous DSBs, these results suggests that the very short deletions are not the consequence of DSB repair but of a different process that is upregulated in the absence of HR proteins.” 

We would like to note that this whole issue is discussed in detail in the discussion section.*

**Second round of review**

**Reviewer 1**

My concerns have been addressed and no more comments.

**Reviewer 2**

Review of revision 1 by Poti et al, concerning SBS3 detection in HR deficient DT40 derivatives.

I thank the reviewers for answering my questions, some of which have been adequately addressed. 
First my main concern; the low level of mutations in the cell lines they analyzed. I thank the authors for clarifying their NMF approach, combining the 3 clones per condition into a single sample for NMF, I had missed that fact previously. Unfortunately it is not the correct analysis design. By combining the 3 clones per condition, the mutation counts don’t go up, so it doesn’t help anything there, and what you lose is the information on biological variation (effectively, by summing the 3 replicates together you average out biological variation). The analyses have to be redone properly, treating the biological replicates as what they are; biological replicates. 
Concerning the analysis the authors included to show the mutation levels suffice (mainly shown in sup. Fig S5); I thank the authors for including this, I think it properly captures NMF’s ability to separate the SBS3 and bg components of the input spectra. 

I also thank the authors for including the analysis using the new set of signatures established by the PCAWG consortium, I think this is important to make sure their results are future-proof. I do see why they prefer not to use the new signature set; it very nicely makes my point; the similarity between their HRD signature and SBS3 and SBS40 seems nearly identical (sup fig S4a); proving my point of the difficulty to distinguish ‘featureless’ signatures from one-another especially if the mutation counts aren’t high enough.
This also links with their signature assignment, where they show SBS3 is present in the majority of ovarian cancers. I know this has been reported before (the authors cite this), but it doesn’t make it true. A tumor with a homozygous BRCA1 or BRCA2 deficiency is expected to show large amounts of associated mutations, so a tumor with 30 mutations assigned to SBS3, is just falsely assigned (again, to distinguish a flat signature such as SBS3 from 5 or 40, you need large amounts of mutations). Signature assignment is not a mathematical exercise, it’s answering a biological question, so biological knowledge about the aetiology of the signatures should be taken into account. Similarly, assigning SBS4 (associated with tobacco smoking) to a skin tumor that is formed on someone’s big toe doesn’t make biological sense. It might look the same, but the obvious biological conclusion would be that it must be a different aetiology, leading to a very similar mutational signature. 

Concerning the indels; the authors have failed to provide the indel mutation spectra I requested; they only put some assignment plot on dropbox, and the supplementary information isn’t in a format that I can use as input for the tools I know are able to generate the indel spectra. Thus I’m unable to properly examine the data underlying the assignments.

**Reviewer 3**

The authors made great efforts to address the potential confounding effects of passage-dependent accumulation. The authors presented pre-existing mutations in the ancestral clones in the new figure S3. They concluded that the majority of pre-existing mutations in all the HR mutants show the HRD spectrum. However, the relative contribution of HRD signature is very different between new mutations in descendent clones and pre-existing mutations in ancestral clones (e.g. ~90% vs 60% in XRCC3 KO cell lines). Did these discrepancies reflect the confounding effects of passage number and cell culture conditions? Additionally, RAD52 KO cells was just created by the authors. Why were there a lot of mutations in ancestral clones in comparison to descendant clones after 50 days of cell culture?

**Authors Response**

**Point-by-point responses to the reviewers’ comments:**

Reviewer #1: My concerns have been addressed and no more comments. 

Reviewer #2: Review of revision 1 by Poti et al, concerning SBS3 detection in HR deficient DT40 derivatives. 

- First my main concern; the low level of mutations in the cell lines they analyzed. I thank the authors for clarifying their NMF approach, combining the 3 clones per condition into a single sample for NMF, I had missed that fact previously. Unfortunately it is not the correct analysis design. By combining the 3 clones per condition, the mutation counts don’t go up, so it doesn’t help anything there, and what you lose is the information on biological variation (effectively, by summing the 3 replicates together you average out biological variation). The analyses have to be redone properly, treating the biological replicates as what they are; biological replicates. 

*We thank the reviewer for this suggestion. We have followed the reviewer’s request and recalculated the NMF using the individual sequenced clones as input sample sets rather than using the genotype-specific sums of mutational events. The results do not change our main conclusions: the HRD spectra derived using the two approaches show a cosine similarity of 0.998, and the BG spectra show a cosine similarity of 0.987. We have included the new data in figure 2A and in the supplementary tables. We have also updated the labelling on the axes of the Fig. 2A panels to clarify the mutation counts used for NMF. 
We have described the new NMF approach in the text and updated the relevant figure legend.* 

- Concerning the analysis the authors included to show the mutation levels suffice (mainly shown in sup. Fig S5); I thank the authors for including this, I think it properly captures NMF’s ability to separate the SBS3 and bg components of the input spectra. 

*Thank you.* 

- I also thank the authors for including the analysis using the new set of signatures established by the PCAWG consortium, I think this is important to make sure their results are future-proof. I do see why they prefer not to use the new signature set; it very nicely makes my point; the similarity between their HRD signature and SBS3 and SBS40 seems nearly identical (sup fig S4a); proving my point of the difficulty to distinguish ‘featureless’ signatures from one-another especially if the mutation counts aren’t high enough. 
This also links with their signature assignment, where they show SBS3 is present in the majority of ovarian cancers. I know this has been reported before (the authors cite this), but it doesn’t make it true. A tumor with a homozygous BRCA1 or BRCA2 deficiency is expected to show large amounts of associated mutations, so a tumor with 30 mutations assigned to SBS3, is just falsely assigned (again, to distinguish a flat signature such as SBS3 from 5 or 40, you need large amounts of mutations). Signature assignment is not a mathematical exercise, it’s answering a biological question, so biological knowledge about the aetiology of the signatures should be taken into account. Similarly, assigning SBS4 (associated with tobacco smoking) to a skin tumor that is formed on someone’s big toe doesn’t make biological sense. It might look the same, but the obvious biological conclusion would be that it must be a different aetiology, leading to a very similar mutational signature. 

*We fully agree with the referee that the appearance of a particular signature in a deconstruction exercise often does not have biological meaning, and that it is important to use only biologically relevant signatures for deconstruction whenever possible (for example, if the underlying mechanism of the particular signatures is known and relevant). Our analysis on signature deconstruction vs mutation numbers, quoted above, indeed supports the referee’s point that assigning SBS3 to samples with few mutations carries a high level of uncertainty. We have therefore removed passages both from the introduction and the results section stating that presence of SBS3 in non-BRCA mutant ovarian cancers suggests HR defects. Instead, we rephrased the introduction to cite and quote a very relevant article (Dong, 2016), which looks at the entire mutation spectrum without signature deconstruction, and also shows mutational burden: “there exists a cohort of non-BRCA mutant ovarian cancer cases with a similar mutation spectrum and mutation burden to BRCA mutants (19), and PARP inhibitor treatment was found to be effective in a patient cohort without germline BRCA mutation as well (20)”* 

- Concerning the indels; the authors have failed to provide the indel mutation spectra I requested; they only put some assignment plot on dropbox, and the supplementary information isn’t in a format that I can use as input for the tools I know are able to generate the indel spectra. Thus I’m unable to properly examine the data underlying the assignments. 

*We apologise for this oversight. We have now included the classification of indels according to PCAWG criteria in the manuscript. The raw classification data is presented in Table S12, and the script used is uploaded at the indicated github site. An indel signature deconstruction using the most dominant signatures present in the dataset is shown on a new supplementary figure (S6). As discussed in the previous round of review, the classification indeed detects ID6 in BRCA2 and PALB2 mutants. We remark on this in the main text: “In agreement with this analysis, a classification of indels into COSMIC version 3 short indel signatures (38) found the microhomology deletion dominated signature ID6 mainly in the BRCA2–/– and PALB2–/– datasets (Fig. S6, Additional File 1).”* 

Reviewer #3: 
- The authors made great efforts to address the potential confounding effects of passage-dependent accumulation. The authors presented pre-existing mutations in the ancestral clones in the new figure S3. They concluded that the majority of pre-existing mutations in all the HR mutants show the HRD spectrum. However, the relative contribution of HRD signature is very different between new mutations in descendent clones and pre-existing mutations in ancestral clones (e.g. ~90% vs 60% in XRCC3 KO cell lines). Did these discrepancies reflect the confounding effects of passage number and cell culture conditions? 

*We can offer a simple explanation for the varying proportions of the two signatures detected amongst pre-existing mutations. As the mutation detection method finds all unique mutations in each sample, it detects mutations in ancestral clones which arose after their lineage had split from the last common ancestor with any of the other cell lines. In case of HR mutants, this last common ancestor predates the HR gene knockout event, prior to which the HR mutant lineage also had an HR-proficient wild type genotype. Therefore in case of the HR mutants we are detecting the combined mutational outcome of two periods of passaging: first as wild type, then as mutant. This is the cause of a higher proportion of BG mutations in the pre-existing mutation sets than in the experimental sets. We inserted a schematic diagram as figure S3B to illustrate this point.* 

- Additionally, RAD52 KO cells was just created by the authors. Why were there a lot of mutations in ancestral clones in comparison to descendant clones after 50 days of cell culture? 

*In concordance with the above argument, the likely reason for this is that we are not only detecting mutations in the RAD52 KO which arose since the KO event, but all those which arose since the last common ancestor. The only way to detect mutations that take place exclusively in the mutant cells is to perform ‘before-and-after’ sequencing according to our experimental plan shown in Fig. 1A. 

To summarise the analysis of pre-existing mutations, it may be sufficient to conclude (as we did in the ms) that the different numbers of pre-existing mutations per cell line suggest different amounts of past passaging, but this in no way correlates with, and therefore does not appear to affect, the identical mutation rates measured under the controlled conditions of our experiments.*

**Third round of review**

**Reviewer 2**

I thank the authors for including the indel catalogs, and making their script to generate these available.
Seeing as classification of indels is non-trivial, I decided to check how the authors handled this problem.

The first problem I encountered is that no example input was provided, making parts of the code difficult to comprehend (e.g. line 8: x$label <- apply(x, 1, function(x) paste0(x[12], "_", x[13]))); if you don't know what's in object x, there's no way to know what the result of this is.
Assuming that supplementary table S3 was a possible input for this code (this did happen to have 13 columns, but the comment on line 16 says "for each genotype/treatment combination separately", while columns 12 and 13 in sup.table S3 are treatment and deletion category, so something is different...), I proceeded.

assuming object x is indeed sup.table S3, the x$label that is created in the code mentioned above is a concatenation of the treatment with a pre-classification of the indels (column 13 in sup.table S3). Therein lies the second problem; how was this subclassification generated? is this a standard classification provided by the Isomut variant caller that was developed at the authors lab? I can't find any mention of this in the methods section.

being curious, I then wanted to check whether this pre-classification (however it was generated) was correct, so I opened the UCSC genome browser, and plugged in a random deletion from sup.table S3 (I picked deletions, because they are simples to identify, as the deleted sequence should be present at the location provided in the table). After some trial and error I was able to with a reasonable certainty conclude that the authors had used the 2011 version of the G.gallus genome (ICGSC Gallus_gallus-4.0/galGal4) for their analyses (as the deleted sequence for 3 different deletions wasn't present at the correct locations in versions 5 and 6 of the g.gallus genome). I suggest the authors add this information to the manuscript (I can't seem to be able to find it...).

The manual checking in the UCSC browser showed the classification in sup.table S3 is correct, so I just ask the authors clarify where this classification came from, as their indel classification script is fully dependent on this.

no further comments.

**Reviewer 3**

The authors have largely addressed my concerns. I don't have further comments.

**Authors Response**

**Point-by-point responses to the reviewers’ comments:**

Reviewer 2

*We now specify that reads were aligned to the Galgal4.73 reference genome. We further clarify that the “COSMIC version 3 short indel classification (Additional File 2: Table S12) was performed using a custom script that used the IsoMut indel output list (Additional File 2: Table S3) as its input.” The deletion preprocessing code is now included in the indel classification script uploaded to github.*
